# Two separate pathways regulate protein stability of ATM/ATR-related protein kinases Mec1 and Tel1 in budding yeast

**DOI:** 10.1371/journal.pgen.1006873

**Published:** 2017-08-21

**Authors:** Greicy H. Goto, Hiroo Ogi, Himadri Biswas, Avik Ghosh, Seiji Tanaka, Katsunori Sugimoto

**Affiliations:** 1 Department of Microbiology, Biochemistry and Molecular Genetics, International Center for Public Health, New Jersey Medical School, Rutgers, The State University of New Jersey, Newark, NJ, United States of America; 2 Division of Microbial Genetics, National Institute of Genetics, and Department of Genetics, School of Life Science, Graduate School for Advanced Studies, (SOKENDAI), Mishima, Shizuoka, Japan; University of California San Francisco, UNITED STATES

## Abstract

Checkpoint signaling requires two conserved phosphatidylinositol 3-kinase-related protein kinases (PIKKs): ATM and ATR. In budding yeast, Tel1 and Mec1 correspond to ATM and ATR, respectively. The Tel2-Tti1-Tti2 (TTT) complex connects to the Rvb1-Rvb2-Tah1-Pih1 (R2TP) complex for the protein stability of PIKKs; however, TTT-R2TP interaction only partially mediates ATM and ATR protein stabilization. How TTT controls protein stability of ATM and ATR remains to be precisely determined. Here we show that Asa1, like Tel2, plays a major role in stabilization of newly synthesized Mec1 and Tel1 proteins whereas Pih1 contributes to Mec1 and Tel1 stability at high temperatures. Although Asa1 and Pih1 both interact with Tel2, no Asa1-Pih1 interaction is detected. Pih1 is distributed in both the cytoplasm and nucleus wheres Asa1 localizes largely in the cytoplasm. Asa1 and Pih1 are required for proper DNA damage checkpoint signaling. Our findings provide a model in which two different Tel2 pathways promote protein stabilization of Mec1 and Tel1 in budding yeast.

## Author summary

We investigated the mechanisms underlying the stability of ATM/ATR-related protein kinase Mec1 and Tel1, which control the DNA damage response in budding yeast. To this end, we applied genetic approaches in combination with a new version of the auxin-inducible degradation (AID) system. Our data are consistent with the model in which two separate pathways regulate protein stability of Mec1 and Tel1, and contribute to proper DNA damage response in budding yeast.

## Introduction

Chromosomes are constantly challenged by exogenous and endogenous threats. The repair of damaged chromosomes is therefore crucial for maintaining genome stability [1–3]. Improper DNA damage response induces genomic instability, resulting in cancer development. The cellular responses to DNA damage consist of DNA repair and checkpoint signaling [4, 5]. Activation of checkpoint signaling depends on two evolutionarily conserved phosphatidylinositol 3-kinase (PI3K)-related protein kinases (PIKKs): ATM and ATR [4, 5]. In the budding yeast *Saccharomyces cerevisiae* ATM and ATR correspond to Tel1 and Mec1, respectively [4]. ATM/Tel1 responds primarily to DNA double-strand breaks (DSBs) [6], whereas ATR/Mec1 recognizes various types of DNA lesions with single-stranded DNA (ssDNA) [7]. ATM is recruited to DSB ends and activated by interacting with the Mre11 complex consisting of Mre11-Rad50-Xrs2 (Nbs1 in human) [8, 9]. ATR forms a stable complex with ATRIP (equivalent to Ddc2 in budding yeast) [10–13], which recruits ATR to sites of DNA damage by interacting with replication protein A (RPA)-coated ssDNA [14–17]. Once activated, ATM and ATR phosphorylate checkpoint mediators (for example, MDC1 in humans and Rad9 in budding yeast) that create a docking site for the effector kinases, such as Chk2 in human and Rad53 in budding yeast [4, 5]. Interaction with checkpoint mediators allows ATM/Tel1 and ATR/Mec1 to extensively phosphorylate the effector kinases, thereby promoting full activation of checkpoint responses [4, 5].

The ATM- and ATR-mediated checkpoint response is under another layer of control besides protein-protein interaction at sites of DNA damage. Several lines of evidence have indicated that the conserved Tel2-Tti1-Tti2 (TTT) complex interacts with and controls protein maturation and stabilization of ATM and ATR family proteins [18–23]. Consistent with this notion, TTT has been shown to play a role in DNA damage response in multiple organisms, including budding yeast [18, 20–22, 24–28]. ATM and ATR family proteins also control telomerase recruitment to telomeres [29–33]. TTT has been implicated in telomere length control in budding yeast cells as well as nematodes [34–37]. TTT connects to the R2TP complex that interacts with the conserved Hsp90 chaperone [23, 38–40]. The R2TP complex consists of four proteins including AAA-type ATPase Rvb1 and Rvb2, Tah1 and Pih1 in budding yeast [38]. The R2TP complex is conserved in eukaryotes; Rvb1, Rvb2, Tah1 and Pih1 correspond to RUVBL1, RUVBL2, PRAP3 and PIH1D1, respectively, in mammals [41]. Rvb1 and Rvb2 associate with each other as a double hexamer to interact with Pih1 in budding yeast and mammalian cells [41]. Recent evidence indicates that Tel2 is constitutively phosphorylated by casein kinase 2 and its phosphorylation mediates Tel2-Pih1 interaction in budding yeast and mammalian cells [23, 40]. Thus, TTT cooperates with the Hsp90 chaperone pathway through the R2TP complex. However, the TTT-R2TP connection does not fully explain TTT-mediated protein stabilization of ATM and ATR. Disruption of Tel2-Pih1 interaction had a clear impact on the protein stability of mTOR and SMG1 but only partially affected the protein stability of ATM and ATR in mouse cells [23]. How TTT promotes the protein stability of ATM and ATR has yet to be defined.

The essential Tra1 protein is homologous to human TRRAP, a protein component of histone acetyltransferase complexes [42]. A combination of serial proteomic approaches has identified a Tra1-containing protein complex, called ASTRA, as a potential chromatin-remodeling factor [43]. In the ASTRA complex, Tra1 interacts with Asa1, the Rvb1-Rvb2 complex and the Tel2-Tti1-Tti2 complex [43]. Although Tra1 does not exhibit protein kinase activities, it shares some structural similarities to PIKKs [42]. Indeed, TTT is required to maintain proper expression of Tra1 protein [28, 44]. Like *tel2* mutations, the *asa1-1* mutation decreases the level of Tel1 and Tor1 protein [37]. Consistent with defective Tel1 and Tor1 expression, *asa1* mutant cells exhibit telomere length defects and sensitivities to rapamycin [37]. Thus, Asa1 has been implicated as a functional partner of TTT in PIKK biogenesis. At this moment, however, it is not known whether Asa1 is incorporated into the TTT-R2TP module.

In this study we show that the TTT complex regulates Mec1 and Tel1 protein stability, and that the Rvb1-Rvb2 complex is essential for proper Mec1 and Tel1 protein expression. We further show that Asa1 controls stability of newly synthesized Mec1 and Tel1 protein whereas Pih1 is required for Mec1 and Tel1 protein stability primarily at high temperatures. Tel2 and Pih1 are localized to both the nucleus and the cytoplasm whereas Asa1 is predominantly located in the cytoplasm. Although both Asa1 and Pih1 interact with Tel2, no Asa1-Pih1 interaction is detected even at high temperatures. Our results support a model in which two different Tel2-mediated pathways control protein stability of Mec1 and Tel1.

## Results

### Tel2 regulates protein stability and functions of Mec1 and Tel1 proteins

**Fig 1:**
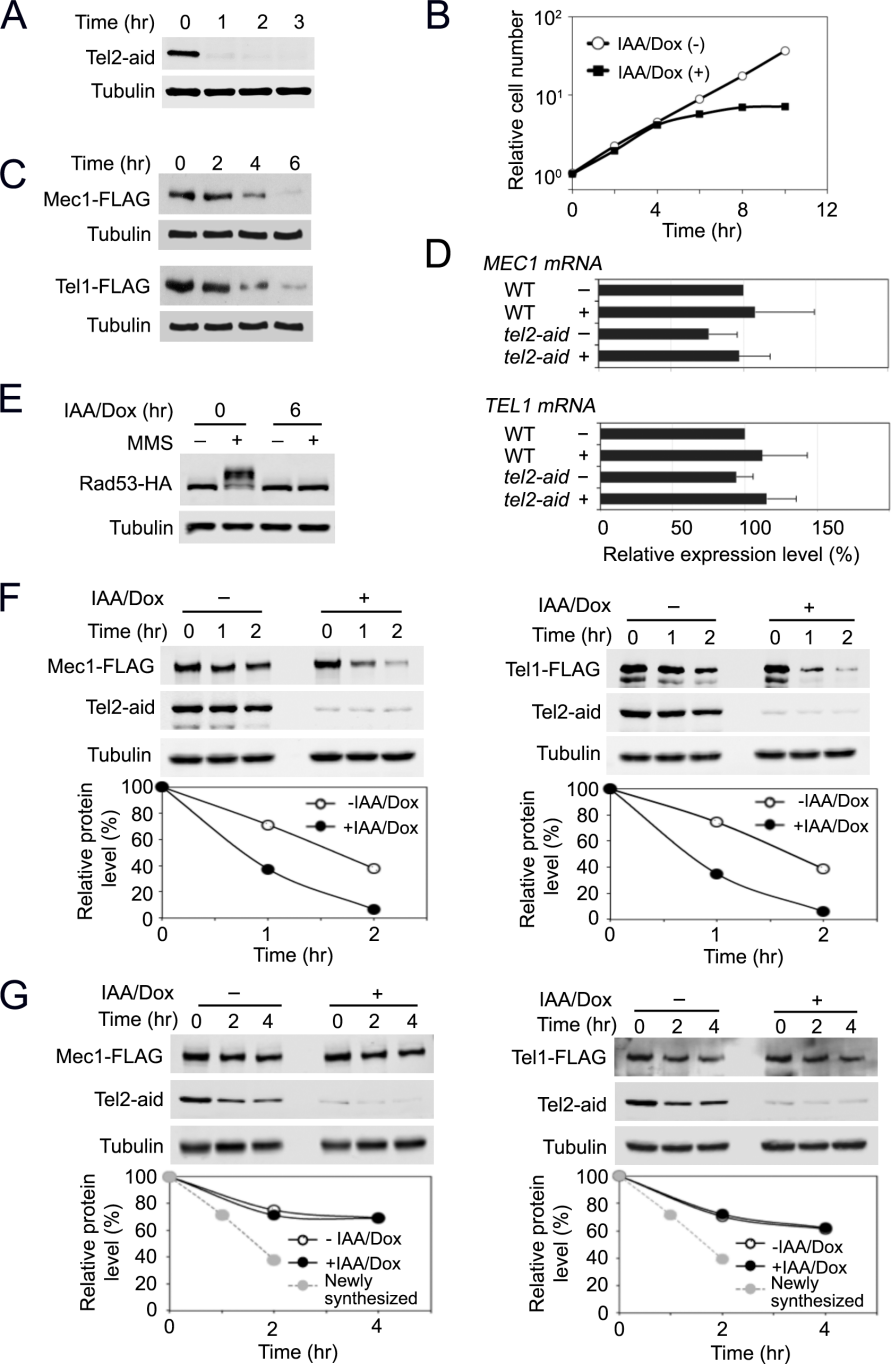
Effect of Tel2 depletion on Mec1 and Tel1 functions. (A) Expression of Tel2-aid after AID activation. Cultures of *tel2-aid* cells were treated with 3-Indoleacetic acid (IAA) and doxycycline (Dox) for the indicated time periods. Cells were analyzed by immunoblotting with anti-AID or anti-tubulin antibodies. (B) Cell proliferation after Tel2 depletion. Cultures of *tel2-aid* cells were treated as in A. Cells were counted using a hematocytometer under a microscope. (C) Expression levels of endogenous Mec1 or Tel1 protein after Tel2 depletion. *tel2-aid* cells expressing Mec1-FLAG or Tel1-FLAG were incubated with IAA and Dox for the indicated time periods. Cells were subjected to immunoblotting analysis with anti-FLAG or tubulin antibodies. (D) Levels of *MEC1* or *TEL1* mRNA after Tel2 depletion. Wild-type or *tel2-aid* cells were mock-treated (-) or incubated with IAA and Dox (+) for 6 hr. Cells were subjected to quantitative PCR analysis to estimate mRNA levels of *MEC1* and *TEL1*. (E) Rad53 phosphorylation after MMS treatment. *tel2-aid* cells expressing Rad53-HA were arrested with nocodazole at G2/M. Half of the cells was further treated with IAA and Dox for 6 hr to deplete Tel2. Cells were exposed to 0.1% MMS for 30 min and analyzed by immunobloting with anti-HA or anti-tubulin antibodies. (F) Stability of newly synthesized Mec1 and Tel1 proteins. *tel2-aid* cells, carrying the GAL-FLAG-MEC1 or the GAL-FLAG-TEL1 plasmid, were grown in sucrose with or without IAA and Dox for 2 hr to deplete Tel2 protein. Galactose was added (to 2% final) to induce Mec1 and Tel1 expression from the *GAL1* promoter. Galactose medium also contained 0.5% or 0.3% glucose to express Mec1 or Tel1 at the endogenous levels, respectively. After 3 hr incubation in galactose, glucose (to 2% final) and cycloheximide (to 10 μg/ml final) were added to shut off new Mec1 and Tel1 protein synthesis (Time point 0 hr). Cells were collected at the indicated times and subjected to immunoblotting analysis with anti-AID, anti-FLAG or anti-tubulin antibodies. (G) Stability of pre-synthesized Mec1 and Tel1 proteins. *tel2-aid* cells, carrying the GAL-FLAG-MEC1 or the GAL-FLAG-TEL1 plasmid, were grown in galactose medium to induce Mec1 and Tel1 expression from the *GAL1* promoter. Cells were then incubated in glucose to turn off the *GAL1* promoter and allow maturation of Mec1 and Tel1 proteins. After 6 hr culture in glucose, Dox/IAA and cycloheximide were added to deplete Tel2 protein and block new protein synthesis (Time point 0 hr). Cells were collected at the indicated times and subjected to immunoblotting analysis with anti-AID, anti-FLAG or anti-tubulin antibodies. The retention curve of the newly synthesized Mec1 or Tel1 protein (without IAA/Dox) is extrapolated from F (a broken gray line).

We adapted an improved auxin-inducible degron (AID) system [45, 46] to further delineate Tel2 function. In this system, target proteins are destroyed by auxin-mediated protein degradation and transcription of target genes is repressed by using the *tetO* promoter [46]. Tel2-aid protein was largely depleted within one hour after the addition of indole-3-acetic acid (IAA) and doxycycline (Dox) (Fig 1A). *TEL2* is essential for cell proliferation [34]. Correspondingly, *tel2-aid* mutants ceased cell proliferation in the presence of IAA and Dox (Fig 1B). Tel2 depletion did not lead to cell-cycle stage specific arrest (S1 Fig), supporting the view that the TTT pathway regulates protein expression of all PIKKs including Tor1 and Tra1 in budding yeast. We first examined the effect of Tel2 depletion on expression levels of Mec1 and Tel1 protein (Fig 1C). Cells expressing FLAG-tagged Mec1 or Tel1 proteins from the respective endogenous promoter were treated with IAA and Dox, and subjected to immunoblotting analysis with anti-FLAG antibodies. Mec1 and Tel1 expression was reduced to less than 15% of the initial level at 6 hr after treatment with IAA and Dox in *tel2-aid* tagged cells but not in untagged cells (Fig 1C and S2 Fig). Quantitative reverse transcription PCR (qRT-PCR) analysis showed that Tel2 depletion does not significantly affect mRNA levels of *MEC1* and *TEL1* (Fig 1D). Extended nocodazole treatment did not decrease levels of Mec1 or Tel1 protein (S3 Fig), supporting that neither prolonged cell-cycle arrest nor proliferation defect affects expression levels of Mec1 and Tel1.

Mec1 and Tel1 both control the DNA damage checkpoint although Mec1 plays a predominant role [4]. Activation of the DNA damage checkpoint pathway is correlated with phosphorylation of the downstream kinase Rad53 [4]. We examined the effect of Tel2 depletion on Rad53 phosphorylation after DNA damage (Fig 1E and S4 Fig). Cells were arrested with nocodazole and treated as above to deplete Tel2 and thereafter exposed to methyl methanesulfonate (MMS). Cells were then analyzed by immunoblotting to monitor Rad53 phosphorylation status. DNA damage-induced Rad53 phosphorylation was significantly decreased after Tel2 depletion. IAA/Dox treatment by itself did not affect damage-induced Rad53 phosphorylation (S5 Fig). Thus, Tel2 plays a key role in activation of DNA damage checkpoint signaling in budding yeast.

We addressed whether Tel2 depletion impairs protein stability of newly-synthesized Mec1 and Tel1 (Fig 1F and S6 Fig). To monitor protein stability, we used *tel2-aid* cells carrying the GAL-FLAG-MEC1 or GAL-FLAG-TEL1 plasmid. We expressed FLAG-tagged Mec1 or Tel1 from the *GAL1* promoter at an expression level similar to the endogenous level. We first depleted Tel2 using the AID system and then transiently induced the expression of Mec1 or Tel1 from the *GAL1* promoter. After glucose and cycloheximide addition (transcription and translation shut-off), we tracked the abundance of Mec1 and Tel1 protein expression to determine the effect of Tel2 depletion on protein stability. Half-lives of newly-synthesized Mec1 and Tel1 protein after transcription and translation shut-off were estimated ~100 min before Tel2 depletion but became ~50 min after Tel2 depletion. Although Tel2-aid was not as stable as tubulin, Tel2-aid protein was present in the presence of cycloheximide if cells were not treated with IAA/Dox.

We next examined whether Tel2 depletion affects protein stability of pre-synthesized Mec1 and Tel1 (Fig 1G and S7 Fig). Cells were initially grown in galactose to express FLAG-Mec1 or Tel1 protein at an expression level similar to the endogenous level and then treated with glucose to repress the *GAL1* promoter. The culture was maintained in glucose for six hours and treated with IAA/Dox or mock-treated for one hour. Cells were subsequently exposed to cycloheximide. We monitored the levels of Mec1 and Tel1 protein Tel2-aid after cycloheximide treatment. Tel2 depletion was found to have no significant impact on protein stability of pre-synthesized Mec1 and Tel1. Half-lives of pre-synthesized Mec1 and Tel1 proteins were estimated longer than 2 hr; pre-synthesized Mec1 and Tel1 proteins are in a stable state compared with newly-synthesized ones.

Together, these results suggest that Tel2 controls protein stability of newly-synthesized Mec1 and Tel1 but does not play an apparent role in the maintenance of pre-synthesized Mec1 and Tel1, supporting the current view that the Tel2-Tti1-Tti2 (TTT) complex promotes protein maturation and regulates functions of ATM and ATR [18, 21, 22].

### Rvb2 is essential to maintain expression levels of Mec1 and Tel1 whereas Pih1 is not

Studies of mammalian cells have provided the model in which the TTT complex interacts with the Rvb1-Rvb2-Tah1-Pih1 (R2TP) complex to recruit the HSP90 chaperone machinery for the proper folding and stabilization of PIKKs [22, 23]. We thus addressed if the R2TP complex mediates TTT-dependent stabilization of Mec1 and Tel1 proteins in budding yeast.

**Fig 2:**
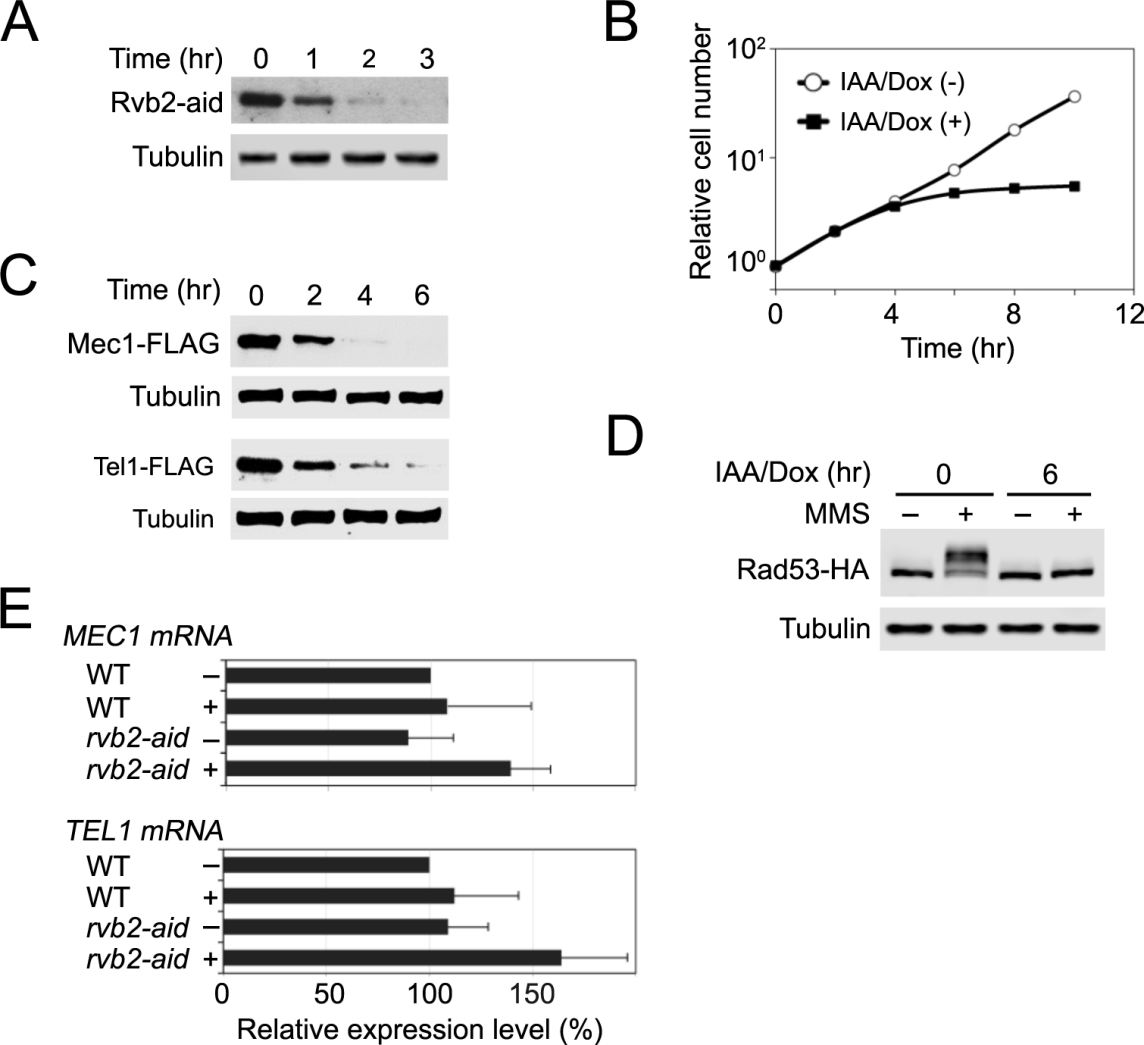
Effect of Rvb2 depletion on Mec1 and Tel1 functions. (A) Expression of Rvb2-aid after AID activation. Cultures of *rvb2-aid* cells were analyzed as in Fig 1A. (B) Cell proliferation after Rvb2 depletion. Cultures of *rvb2-aid* cells were monitored as in Fig 1B. (C) Expression levels of endogenous Mec1 or Tel1 protein after Rvb2 depletion. *rvb2-aid* cells expressing Mec1-FLAG or Tel1-FLAG were analyzed as in Fig 1C. (D) Rad53 phosphorylation after Rvb2 depletion. *rvb2-aid* cells expressing Rad53-HA were examined as in Fig 1E. (E) Levels of *MEC1* or *TEL1* mRNA after Rvb2 depletion. *rvb2-aid* cells were analyzed as in Fig 1D.

We first examined whether the Rvb1-Rvb2 complex controls protein stability of Mec1 and Tel1 as TTT does (Fig 2). Both *RVB1* and *RVB2* are essential for cell proliferation [47, 48]. We generated an *rvb2-aid* allele to examine the role of Rvb1-Rvb2 complex in protein stabilization of Mec1 and Tel1. Rvb2-aid was depleted within two hours after IAA and Dox treatment (Fig 2A). Accordingly, Rvb2 depletion decreased cell proliferation (Fig 2B). No cell-cycle specific arrest resulted from Rvb2 depletion (S8 Fig) [49]. As found for Tel2 depletion, Rvb2 depletion lowered endogenous expression levels of Mec1 and Tel1 (Fig 2C) and impaired Rad53 phosphorylation after MMS treatment (Fig 2D and S9 Fig). The Rvb1-Rvb2 complex associates with chromatin remodeling complexes and controls transcription of numerous genes [43, 50]. Previous genome-wide analyses, however, did not identify *MEC1* and *TEL1* as a transcriptional target gene of Rvb1-Rvb2 complex [49]. We confirmed that Rvb2 depletion does not affect mRNA levels of *MEC1* and *TEL1* by qRT-PCR analyses (Fig 2E). These results are consistent with the model in which the Rvb1-Rvb2 complex collaborates with the TTT complex and contributes to protein stabilization of Mec1 and Tel1. We were not able to carry out the experiment using the GAL-FLAG-MEC1 or GAL-FLAG-TEL1 plasmid, since *RVB1* and *RVB2* are required for induction of the *GAL* promoters [49].

**Fig 3:**
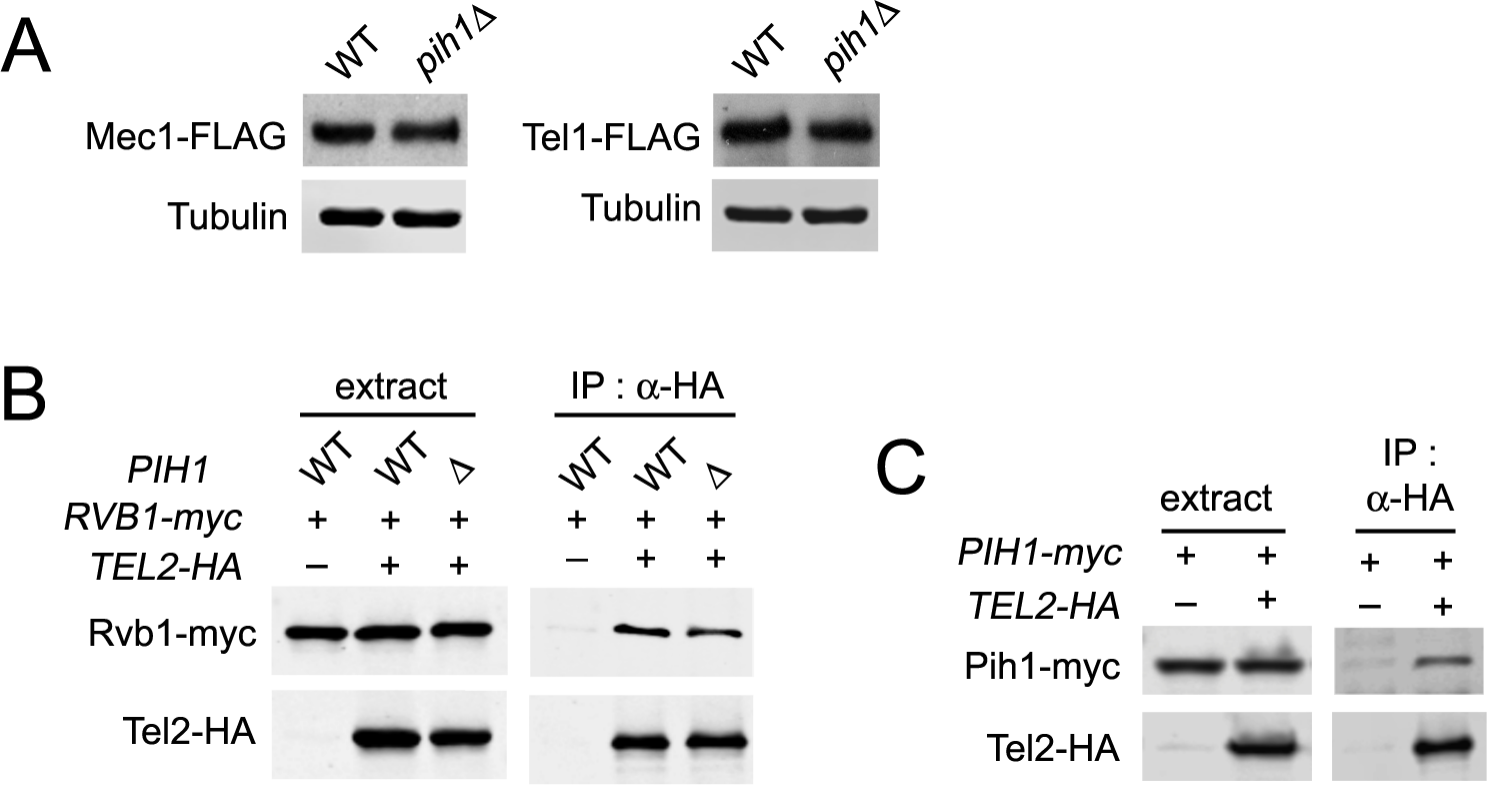
Effect of *pih1* deletion on Mec1 and Tel1 functions. (A) Expression levels of endogenous Mec1 or Tel1 protein. Wild-type and *pih1*Δ cells expressing Mec1-FLAG or Tel1-FLAG were cultured and subjected to immunoblotting analysis with anti-FLAG or anti-tubulin antibodies. (B) Effect of *pih1* deletion on Rvb1-Tel2 interaction. Wild-type and *pih1*Δ cells expressing Tel2-HA or Rvb1-myc were subjected to immunoprecipitation with anti-HA antibodies. Extracts and immunoprecipitates were analyzed by immunoblotting with anti-HA or anti-myc antibodies. (C) Pih1-Tel2 interaction. Cells expressing Pih1-myc or Tel2-HA were analyzed as in B.

We next examined if Pih1 controls expression levels of Mec1 and Tel1 (Fig 3). We note that *PIH1* is not essential for cell proliferation. Deletion in *PIH1* had very minor effects on protein expression levels of Mec1 and Tel1 protein; no apparent or very minor (~10%) decrease was observed for Mec1 or Tel1 protein, respectively (Fig 3A). Pih1/PIH1D1 has been proposed to connect the TTT complex to the Rvb1-Rvb2 complex [23, 40]. However, Rvb1-Tel2 interaction was still detectable even in the absence of Pih1 (Fig 3B). We confirmed that Tel2 interacts with Pih1 by co-immunoprecipitation analysis (Fig 3C). Thus, although the TTT-R2TP axis exists in budding yeast, Pih1-independent mechanisms appear to tether TTT to the Rvb1-Rvb2 complex.

### Asa1 interacts with the Rvb1-Rvb2 complex and stimulates TTT to recognize Mec1 and Tel1

**Fig 4:**
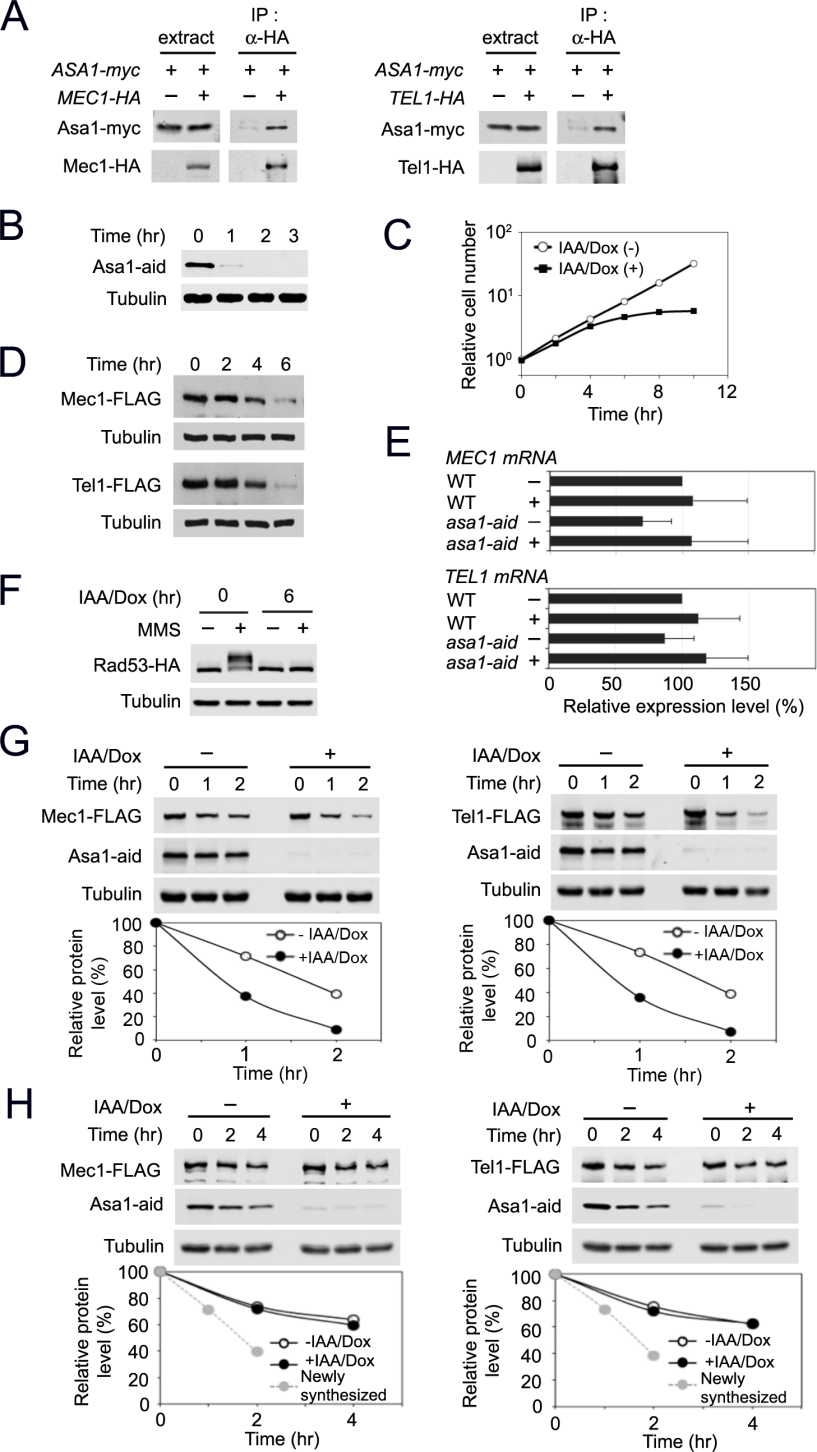
Effect of Asa1 depletion on Mec1 and Tel1 functions. (A) Interaction of Asa1 with Mec1 and Tel1. Cells expressing Asa1-myc and Mec1-HA or Tel1-HA were analyzed as in Fig 3B. (B) Expression of Asa1-aid after AID activation. Cultures of *asa1-aid* cells were analyzed as in Fig 1A. (C) Cell proliferation after Asa1 depletion. Cultures of *asa1-aid* cells were examined as in Fig 1B. (D) Expression levels of endogenous Mec1 or Tel1 protein after Asa1 depletion. *asa1-aid* cells expressing Mec1-FLAG or Tel1-FLAG were analyzed as in Fig 1C. (E) Levels of *MEC1* or *TEL1* mRNA after Asa1 depletion. *asa1-aid* cells were analyzed as in Fig 1D. (F) Rad53 phosphorylation after Asa1 depletion. *asa1-aid* cells expressing Rad53-HA were examined as in Fig 1E. (G) Effect of Asa1 depletion on newly synthesized Mec1 and Tel1 proteins. *asa1-aid* cells, carrying the GAL-FLAG-MEC1 or the GAL-FLAG-TEL1 plasmid, were cultured and analyzed as in Fig 1F. (H) Effect of Asa1 depletion on pre-synthesized Mec1 and Tel1 proteins. *asa1-aid* cells, carrying the GAL-FLAG-MEC1 or the GAL-FLAG-TEL1 plasmid, were cultured and analyzed as in Fig 1G.

Since Asa1 has been implicated as a functional partner of TTT in PIKK biogenesis [37], we next explored the link of Asa1 to TTT-mediated Mec1 and Tel1 protein stabilization. We first examined whether Asa1 interacts with Mec1 and Tel1 by co-immunoprecipitation analysis. Mec1 and Tel1 were co-immunoprecipitated with Asa1 only when cells carried *ASA1-myc* and *MEC1-HA* or *TEL1-HA*, indicating that Asa1 interacts with Mec1 and Tel1 (Fig 4A). We addressed whether Asa1 regulates protein expression of Mec1 and Tel1 at a post-translational level. Similar to Tel2 and Rvb2, Asa1 is essential for cell proliferation [51]. We thus constructed an *asa1-aid* allele and determined the effect of Asa1 depletion on Mec1 and Tel1 protein levels. Asa1 was depleted within one hour after treatment with IAA and Dox (Fig 4B), and Asa1 depletion impaired cell proliferation (Fig 4C). Asa1 depletion did not result in cell-cycle stage specific arrest (S10 Fig). As found for Rvb2 and Tel2 depletion, Asa1 depletion decreased the endogenous protein levels of Mec1 and Tel1 (Fig 4D) but did not affect the transcript levels (Fig 4E). Asa1 depletion was found to impair Rad53 phosphorylation after DNA damage (Fig 4F and S11 Fig).

We addressed whether Asa1 depletion impairs protein stability of newly-synthesized Mec1 and Tel1 (Fig 4G). *asa1-aid* cells, carrying the GAL-FLAG-MEC1 or GAL-FLAG-TEL1 plasmid, were cultured and analyzed as were *tel2-aid* cells (see Fig 1). Asa1 depletion was found to reduce levels of newly-synthesized Mec1 and Tel1 protein (Fig 4G). We next examined whether Asa1 depletion has impact on stability of pre-synthesized Mec1 and Tel1 (Fig 4H). There was no apparent effect of Asa1 depletion on pre-synthesized Mec1 and Tel1 proteins (Fig 4H). Thus, like Tel2, Asa1 appears to control protein stability of newly synthesized Mec1 and Tel1.

**Fig 5:**
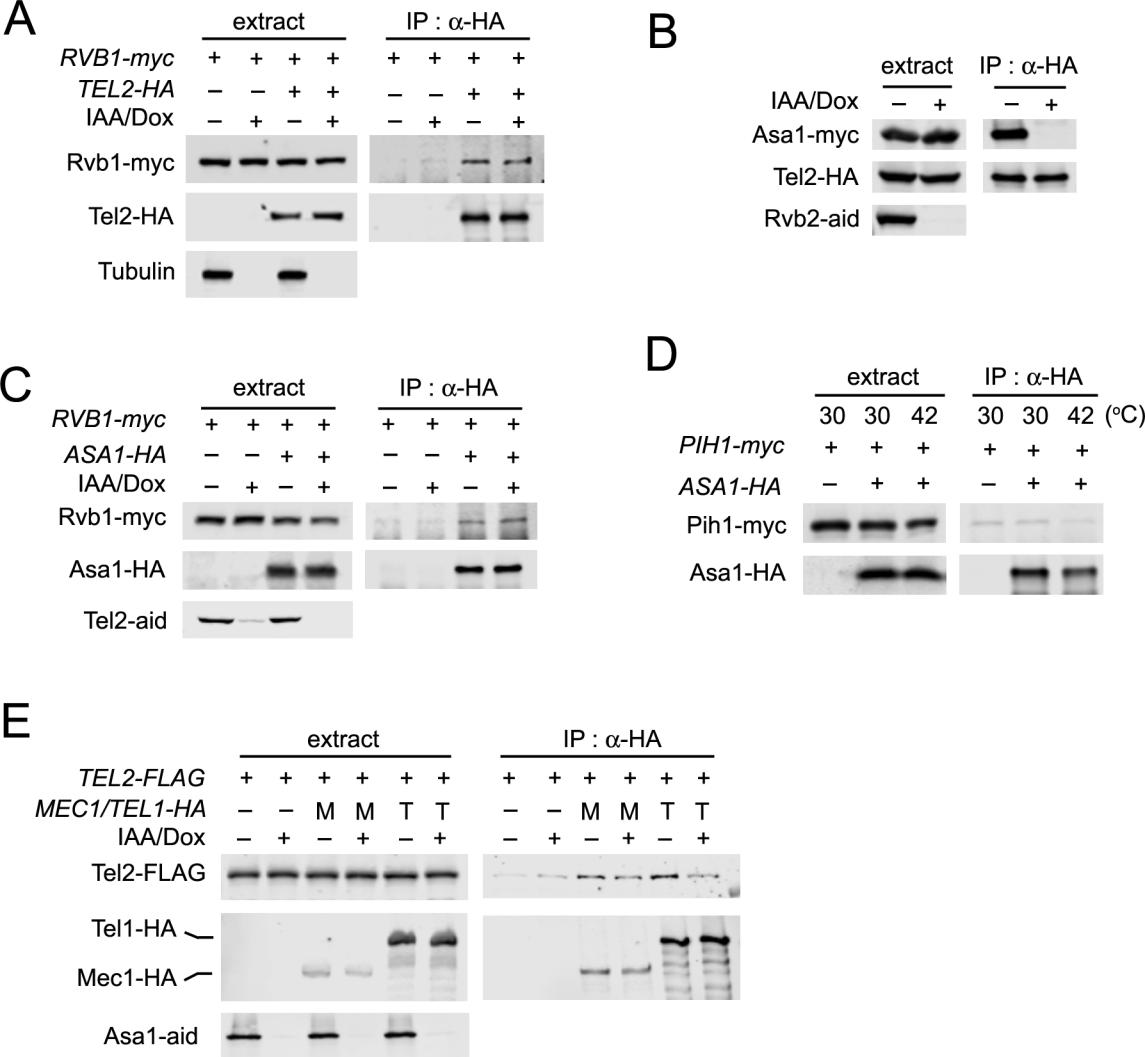
Role of Asa1 in the TTT-R2 pathway. (A) Effect of Asa1 depletion on Rvb1-Tel2 interaction. Cultures of *asa1-aid* cells expressing Rvb1-myc or Tel2-HA were treated with IAA and Dox for 6 hr. Extracts were subjected to immunoprecipitation with anti-HA antibodies. Extracts and immunoprecipitates were analyzed by immunoblotting with anti-AID, anti-HA or anti-myc antibodies. (B) Effect of Rvb2 depletion on Asa1-Tel2 interaction. Cultures of *rvb2-aid* cells expressing Asa1-myc and Tel2-HA were analyzed as in A. (C) Effect of Tel2 depletion on Asa1-Rvb1 interaction. Cultures of *tel2-aid* cells expressing Asa1-HA or Rvb1-myc were analyzed as in A. (D) Co-immunoprecipitation analysis for Asa1-Pih1 interaction at 30°C and 42°C. Cells expressing Asa1-HA or Pih1-myc were cultured at 30°C and subjected to immunoprecipitation with anti-HA antibodies. Cells expressing Asa1-HA and Pih1-myc were transferred to 42°C for 1 hr and subjected to immunoprecipitation with anti-HA antibodies. Extracts and immunoprecipitates were analyzed by immunoblotting with anti-HA or anti-myc antibodies. Note that the background level of Pih1-myc was detected in the immunocomplex from the untagged *ASA1* strain. (E) Effect of Asa1 depletion on Mec1-Tel2 and Tel1-Tel2 interaction. Cultures of *asa1-aid* cells expressing Tel2-FLAG, Mec1-HA (M) or Tel1-HA (T) were treated with IAA and Dox for 2 hr and subjected to immunoprecipitation with anti-HA antibodies. Extracts or immunoprecipitates were analyzed by immunoblotting with anti-AID, anti-FLAG or anti-HA antibodies.

Asa1 is highly conserved in eukaryotes [43], although its molecular function is unknown. Since Rvb1-Tel2 interaction occurs in the absence of Pih1 (see Fig 3B), we considered the possibility that Asa1 mediates the interaction between TTT and the Rvb1-Rvb2 complex (Fig 5). *asa1-aid* cells expressing HA-tagged Tel2 or myc-tagged Rvb1 were treated with or without IAA and Dox. Cells were then subjected to co-immunoprecipitation and subsequent immunoblotting analysis. Unexpectedly, however, Asa1 depletion did not affect Rvb1-Tel2 interaction (Fig 5A). We then examined whether Asa1 associates with either the TTT or the Rvb1-Rvb2 complex. Rvb2 depletion disrupted Asa1-Tel2 interaction (Fig 5B) whereas Tel2 depletion did not affect Asa1-Rvb1 interaction (Fig 5C). These results show that Asa1 interacts with the Rvb1-Rvb2 complex rather than the TTT complex. To address the possibility that Asa1 associates with the R2TP complex, we examined whether Pih1 and Asa1 interact with each other. No apparent interaction between Asa1 and Pih1 was detected (Fig 5D) although both Asa1 and Pih1 are connected to Tel2 (Figs 3C and 5B).

TTT recognizes PIKKs for protein stabilization [18, 21, 22]. We next addressed whether Asa1 contributes to TTT recognition of Mec1 and Tel1. We investigated the effect of Asa1 depletion on Tel2-Mec1 and Tel2-Tel1 interaction (Fig 5E). Two-hour incubation with IAA and Dox largely eliminated Asa1 expression but did not lower the expression levels of Mec1 and Tel1; (Fig 5E; see also Fig 4B and 4D). We note that two-hour Asa1 depletion in this experiment might not be as complete as six-hour depletion used in Fig 5A. Asa1 depletion was found to decrease interaction of Tel2 with Mec1 and Tel1 (Fig 5E). Reduction in Tel2-Tel1 interaction was more apparent than that in Tel2-Mec1 interaction (Fig 5E). These results suggest that Asa1 interacts with the Rvb1-Rvb2 complex and stimulates TTT to recognize Mec1 or Tel1 protein.

### Pih1 contributes to protein stability of Mec1 and Tel1 at high temperatures

**Fig 6:**
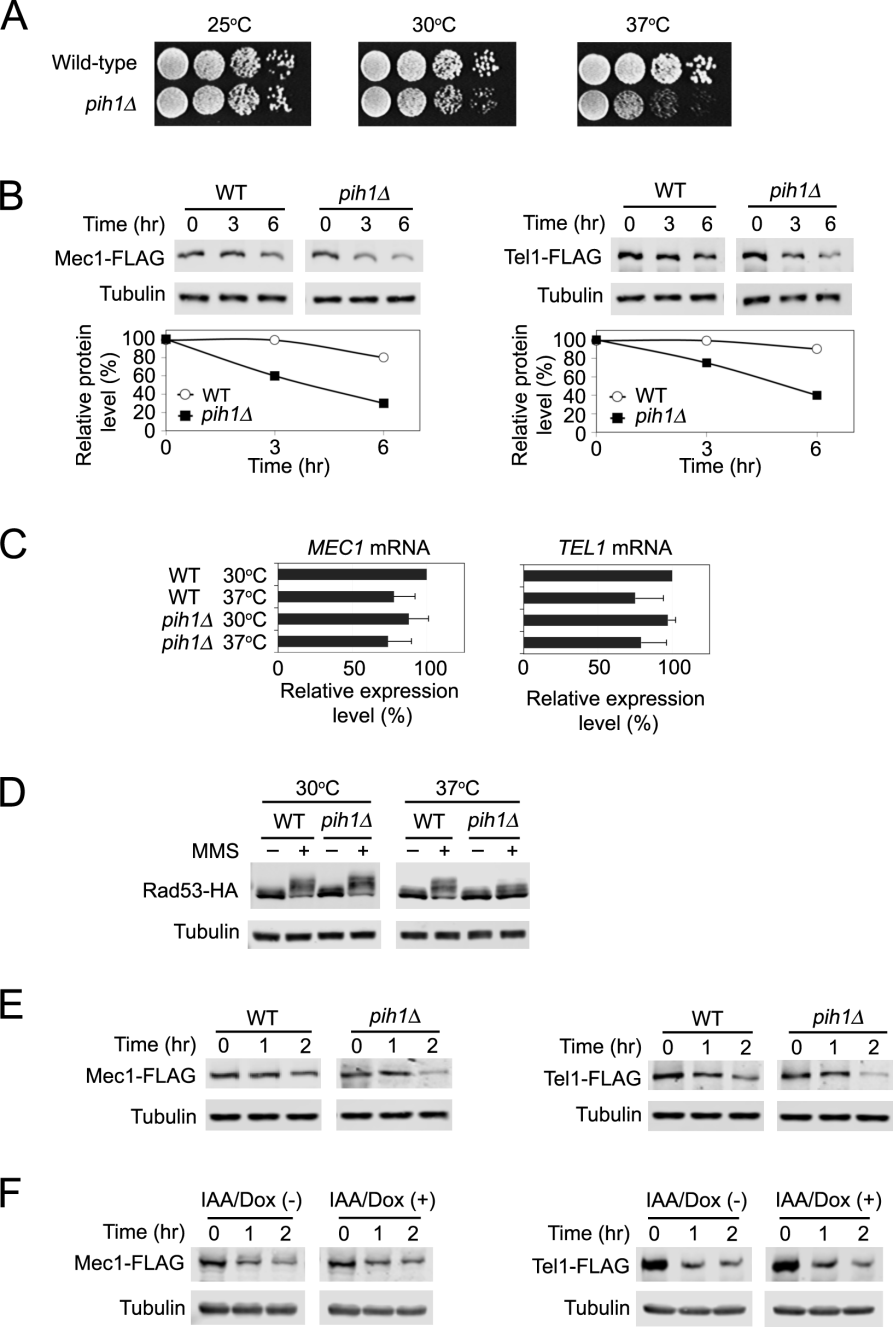
Role of Pih1 in protein stabilization of Mec1 and Tel1 at high temperatures. (A) Temperature sensitivity of *pih1Δ* mutants. Ten-fold serial dilutions of cultures were spotted on YEPD medium. Plates were incubated at 25, 30 or 37°C for 2~3 days. (B) Effect of *pih1* deletion mutation on endogenous Mec1 and Tel1 protein levels at high temperatures. Wild-type and *pih1Δ* cells expressing Mec1-FLAG or Tel1-FLAG were grown at 30°C and transferred to 37°C for the indicated times. Cells were subjected to immunoblotting analysis with anti-FLAG or anti-tubulin antibodies. (C) Effect of *pih1* deletion mutation on *MEC1* and *TEL1* mRNA levels at high temperatures. Wild-type and *pih1Δ* cells were grown at 30°C and then transferred to 37°C for 3 hr. Cells were analyzed as in Fig 1D. (D) Effect of *pih1* deletion on Rad53 phosphorylation. Wild-type and *pih1*Δ cells expressing Rad53-HA were arrested with nocodazole and exposed to MMS at 30°C (left panel). Arrested cells were also transferred to 37°C for 6 hr and exposed to MMS (right panel). Cells were then analyzed by immunobloting with anti-HA or anti-tubulin antibodies. (E) Effect of *pih1Δ* mutation on pre-synthesized Mec1 and Tel1 protein stability at high temperatures. Wild-type and *pih1*Δ cells, carrying the GAL-FLAG-MEC1 or the GAL-FLAG-TEL1 plasmid, were initially grown in galactose medium to induce Mec1 and Tel1 expression from the *GAL1* promoter at 30°C. Cells were then incubated in 2% glucose to turn off the *GAL1* promoter and allow protein maturation of Mec1 and Tel1 at 30°C. After 6 hr incubation with glucose, cultures were treated with cycloheximide and transferred to 42°C (Time point 0 hr). Cells are collected at the indicated time and examined by immunoblotting with anti-FLAG or tubulin antibodies. (F) Effect of Asa1 depletion on pre-synthesized Mec1 and Tel1 protein stability at high temperatures. *asa1-aid* cells, carrying the GAL-FLAG-MEC1 or the GAL-FLAG-TEL1 plasmid, were initially grown in galactose medium to induce Mec1 and Tel1 expression from the *GAL1* promoter at 30°C. Cells were then incubated in 2% glucose to turn off the *GAL1* promoter and allow protein maturation of Mec1 and Tel1 at 30°C for 6 hr. Cultures were then treated with Dox/IAA for one hour and subsequently transferred to 42°C or retained at 30°C (at time point 0 hr). Cells are collected at the indicated time and examined by immunoblotting with anti-FLAG or tubulin antibodies. We note that cells were treated as in Figs 1G and 4H before “time point” 0 hr (See S7 and S17 Figs).

We explored the role of Pih1 in Mec1 and Tel1 protein stability (Fig 6). Although *PIH1* is not essential for cell proliferation, *pih1* deletion confers temperature-sensitive growth defects (Fig 6A) [40]. We therefore tested a possibility that Pih1 contributes to Mec1 and Tel1 protein stabilization at high temperatures. We examined the effect of *pih1Δ* mutation on Mec1 and Tel1 protein levels after transferring from 30 to 37°C (Fig 6B). Deletion of *PIH1* decreased expression levels of Mec1 and Tel1 proteins at 37°C (Fig 6B) although it did not significantly affect mRNA levels (Fig 6C). We further examined the effect of *pih1Δ* mutation on DNA damage checkpoint response. The *pih1Δ* mutation conferred a defect in Rad53 phosphorylation after MMS treatment at 37°C although no apparent phosphorylation defect was observed at 30°C (Fig 6D and S12 Fig).

Treatment with cycloheximide was found to stabilize Mec1 and Tel1 proteins at high temperatures (S13 Fig) probably because ubiquitin becomes limiting after translation inhibition [52]. We were therefore unable to use cycloheximide to monitor protein stabilization of Mec1 and Tel1 at high temperatures. Instead we took advantage of the fact that mRNAs are short-lived and most half-lives are 30 min or shorter [53, 54]. If transcripts were absent, the effect of translation would be essentially eliminated. As mentioned above, transcription was shut off for 6 hr to generate pre-synthesized Mec1 and Tel1 proteins. We thus addressed whether Pih1 contributes to stabilization of pre-synthesized Mec1 and Tel1 proteins at high temperatures (Fig 6E and S14 Fig). Wild-type and *pih1Δ* cells carrying the GAL-FLAG-MEC1 or GAL-FLAG-TEL1 plasmid were grown in the presence of galactose to activate the *GAL1* promoter at 30°C and then incubated with glucose to repress the *GAL1* promoter. Cells were cultured in glucose for 6 hr to allow protein stabilization of Mec1 and Tel1. After the incubation with glucose, cultures were transferred to 42°C or retained at 30°C. Deletion of *PIH1* decreased the expression levels of Mec1 and Tel1 after a shift from 30 to 42°C (Fig 6E). No apparent effect on Mec1 and Tel1 expression was detected at 30°C (S15 Fig), supporting the findings that Pih1 is dispensable for proper Mec1 and Tel1 expression at 30°C (see Fig 3A). We confirmed that mRNAs from GAL-FLAG-MEC1 or GAL-FLAG-TEL1 were decayed out before the transfer from 30°C to 42°C (S16 Fig). These results are consistent with the idea that Pih1 controls protein stability of mature Mec1 and Tel1 proteins primarily at high temperatures.

We next investigated the effect of Asa1 depletion on pre-synthesized Mec1 and Tel1 proteins at 42°C (Fig 6F and S17 Fig). *asa1-aid* cells carrying the GAL-FLAG-MEC1 or GAL-FLAG-TEL1 plasmid were grown in the presence of galactose to activate the *GAL1* promoter at 30°C and then incubated with glucose to turn off the promoter and allow protein maturation for 6 hr. Cultures were treated with IAA and Dox or mock-treated for one hour and then transferred to 42°C or retained at 30°C. Asa1 depletion did not significantly affect protein stability of pre-synthesized Mec1 and Tel1 proteins at 42°C as found at 30°C (Fig 6F and S18 Fig; see Fig 4H). Transcripts from the GAL-FLAG-MEC1 or GAL-FLAG-TEL1 construct were essentially at the background level before the temperature shift (S19 Fig). Thus, Asa1 does not appear to play a major role in Mec1 and Tel1 stabilization at high temperatures although it remains possible that Asa1 plays a minor or overlapping role. Asa1-Pih1 interaction was undetectable even at 42°C (Fig 5D). These findings suggest that Asa1 and Pih1 control protein stability of Mec1 and Tel1 at different levels.

### Asa1 localizes largely to the cytoplasm whereas Pih1 is distributed in both the cytoplasm and nucleus

**Fig 7:**
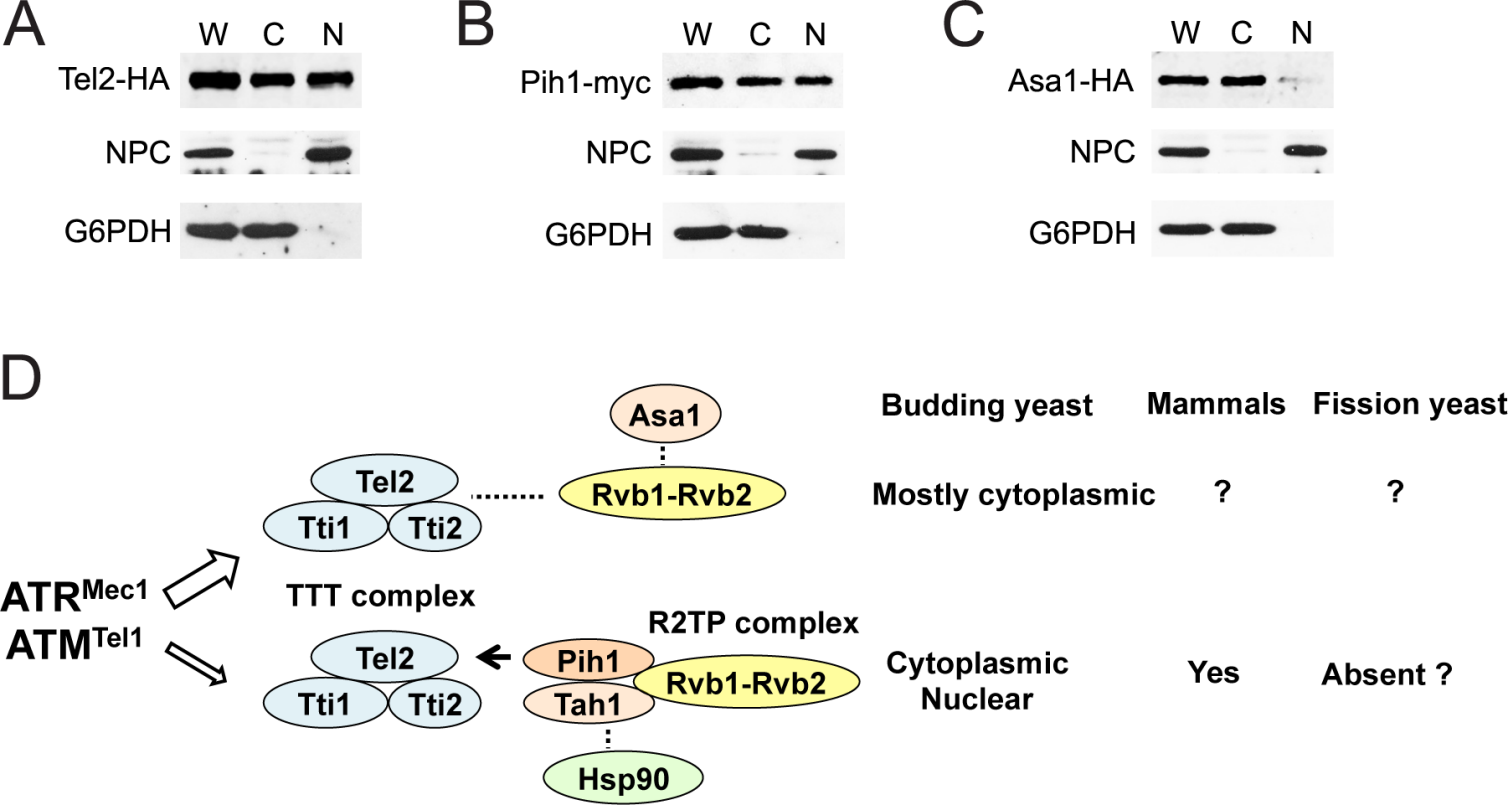
Cellular localization of Asa1 and Pih1. (A, B, C) Cells expressing Tel2-HA (A), Pih1-myc (B) or Asa1-HA (C) were grown to mid log-phase and spheroplasted. Spheroplasts were homogenized to prepare whole-cell extracts (W) and then separated into the cytoplasmic (C) and nuclear (N) fractions. Samples from each fraction were separated by SDS-PAGE and immunoblotted with anti-HA, anti-myc, anti-Zwf1 (Glucose-6-Phosphate Dehydrogenase; G6PDH) or anti-nuclear pore complex (NPC) antibodies. (D) Two different Tel2 pathways and protein localization. See the text.

Mec1 and Tel1 are nuclear proteins although some Mec1 and Tel1 proteins are present in the cytoplasm [12, 55]. To further dissect Asa1 and Pih1 functions, we compared cellular localization of Asa1, Pih1 and Tel2 (Fig 7). Cellular fractionation analysis indicated that Pih1 and Tel2 both exist in both nuclear and cytoplasmic fractions (Fig 7A and 7B). By contrast, Asa1 was largely localized in the cytoplasm (Fig 7C). Together, our results support the model in which the Asa1 and the Pih1 pathways contribute differently to stabilization of protein kinases Mec1 and Tel1 (Fig 7D).

## Discussion

The TTT complex is a key component to ensure proper protein levels of PIKKs including ATM and ATR [18–21]. The R2TP complex, consisting of AAA-ATPase Rvb1 and Rvb2 as well as Tah1 and Pih1, is highly conserved from yeast to humans [41]. Previous studies have demonstrated that casein-kinase-mediated Tel2 phosphorylation promotes Tel2-Pih1 interaction, thereby connecting TTT to R2TP for stabilization of PIKKs [23, 40]. However, mechanisms other than the TTT-R2TP pathway appear to control TTT-dependent functions, because defective Tel2-Pih1/PIH1D1 interaction has much less impact on the stability of ATM and ATR than complete loss of Tel2 function does [23]. In this study we have provided evidence indicating that two different pathways, the Tel2-Pih1 and the Tel2-Asa1 pathway, contribute to the quality control of Mec1 and Tel1 proteins in budding yeast. Like Tel2, Asa1 plays a major role in proper Mec1 and Tel1 protein expression. In contrast, Pih1 is primarily required for Mec1 and Tel1 protein stabilization at high temperatures. Asa1 is largely located in the cytoplasm whereas Pih1 is distributed throughout the cell. It has been shown that Tel2 preferentially recognizes newly synthesized ATM and ATR under non-stress conditions [22]. Our results suggest the model in which the Tel2-Asa1 pathway promotes protein folding of newly synthesized Mec1 and Tel1 in the cytoplasm whereas the Tel2-Pih1 pathway stimulates protein refolding during heat stress.

Studies of mammalian TTT complex have demonstrated that TTT regulates DNA damage signaling as well as ATM and ATR protein stability [18, 21, 22]. In this work we applied an auxin-induced protein degradation (AID) system and confirmed that the TTT pathway is critical for DNA damage checkpoint in budding yeast as well, providing a unified view that TTT-mediated control is conserved from yeast to humans. Depletion of Tel2, Rvb2 and Asa1 caused nearly complete defects in damage-induced Rad53 phosphorylation although there were detectable levels of Mec1 and Tel1 proteins. One explanation could be that the TTT pathway not only stabilizes Mec1 and Tel1 protein but also facilitates interaction of Mec1 and Tel1 with other checkpoint proteins. It has been shown that Tel2 (HCLK2) is required for efficient ATR-TopBP1 interaction and TopBP1-mediated ATR activation in human cells [27]. Supporting this view, previous studies have shown that low levels of Tti1 delocalize Tra1 and Mec1 outside of the nucleus [28].

The R2TP complex is found in organisms from yeast to humans; R2TP consists of Rvb1, Rvb2, Tah1, and Pih1 in budding yeast [41]. Like Tel2 depletion, Rvb2 depletion had a high impact on Mec1 and Tel1 protein expression. The Rvb1-Rvb2 complex interacts with and regulates chromatin-modeling complexes; therefore, dysfunction affects transcription of numerous genes [50]. In humans, knockdown of Rvb1/RUVBL1 or Rvb2/RUVBL2 affects mRNA levels of PIKKs [56]. Rvb2 depletion was not found to affect mRNA levels of *MEC1* and *TEL1*, supporting the idea that Tel2 and Rvb1-Rvb2 constitute a pathway for protein stabilization in budding yeast. The observation that Rvb2 depletion causes defective Rad53 phosphorylation is consistent with a model in which the Rvb1-Rvb2 complex acts in the TTT-mediated PIKK stabilization pathway. However, the observed decreased Rad53 phosphorylation could result at least in part from defective DNA damage repair. Ino80- and Swr1-chromatin remodeling complexes, containing the Rvb1-Rvb2 complex, have been implicated in chromatin remodeling at sites of DNA damage or DNA damage checkpoint signaling in budding yeast [50, 57]. Although our results show that the Rvb1-Rvb2 complex modulates the TTT-Asa1 pathway as well, the exact role of the Rvb1-Rvb2 complex in this pathway remains to be determined.

Newly synthesized polypeptide chains must fold and assemble into specific three-dimensional structures in order to become fully functional. In many cases efficient folding depends on assistance from proteins known as molecular chaperones [58]. Several lines of evidence show that TTT acts as a co-chaperone for Hsp90. Tah1 has been suggested to connect TTT to the Hsp90 chaperone [23, 39]. At this moment it is not clear whether Asa1 collaborates with Hsp90 in budding yeast. Previous systematic approaches identified Pih1 and Tah1 as an Hsp90 interacting protein but did not pick Asa1 out [38]. Hsp90 may interact only transiently or weakly with the TTT-Asa1-Rvb1-Rvb2 complex in budding yeast although it is formally possible that the TTT-Asa1-Rvb1-Rvb2 complex acts independently of Hsp90 protein. Tel2 has been shown to recognize ATM and ATR in an Hsp90-dependent manner in human cells [22]. We found that Tel2 interacts with Mec1 and Tel1 in an Asa1-dependent manner. Asa1 might mediate Hsp90-chaperone functions in collaboration with the Rvb1-Rvb2 complex. Tel2 has been shown to interact with the N-terminal HEAT repeat region of ATM and mTOR *in vitro* [18]. Since the sequence similarity at the N-terminal region of PIKKs is relatively low compared with that at the C-terminal catalytic domain [59], the TTT pathway is expected to process PIKKs with different efficiencies. We found that Asa1 depletion had a more significant impact on Tel2-Tel1 interaction than Tel2-Mec1 interaction. Since Mec1 and Tel1 do not share significant amino acid sequence similarities in the N-terminal region, TTT could interact with Mec1 and Tel1 with different affinities. However, Asa1 might make TTT a good fit for Mec1 and Tel1.

We have provided evidence indicating that Asa1 forms a complex with TTT, which is different from the TTT-R2TP complex in budding yeast. It thus seems likely that the TTT-Asa1 pathway operates separately from the Tel2-Pih1 pathway although we cannot exclude the possibility that these two pathways act redundantly. Pih1 appears to connect TTT to the Rvb1-Rvb2 complex in budding yeast [23, 40] but Pih1 does not appear to exist in the TTT-Asa1-Rvb1-Rvb2 complex. Budding yeast may contain another yet-to-be identified protein that mediates interaction of TTT with the Rvb1-Rvb2 complex. Alternatively, the Rvb1-Rvb2 complex may exert a different mode in which it interacts directly with TTT. In mammals, Tel2-Pih1/PIH1D1 interaction does not fully contribute to protein stabilization of ATM and ATR. Therefore, TTT has been suggested to connect ATM and ATR to Hsp90 independently of PIH1D1. Casein kinase 2 phosphorylates Tel2 in fission yeast as well [60]. Curiously, however, Tel2 phosphorylation is dispensable for TTT-mediated PIKK biogenesis [60]. Pih1 and Tah1 homologs have not been identified in the fission yeast *Schizosaccharomyces pombe* [60, 61]. Thus, TTT appears to control protein stability of ATM and ATR family proteins through several different mechanisms in eukaryotes (Fig 7D). Asa1 is conserved from yeast to humans; an Asa1 homolog has been identified in fission yeast as well [43]. It is interesting to see whether other eukaryotes utilize the Tel2-Asa1 pathway to regulate protein stability of ATM- and ATR-related protein kinases.

## Materials and methods

### Strains, plasmids and cultures

Strains carrying the improved AID system were generated as described [46]. To prepare the AID host strain, the tTA-TetR’-SSN6-OsTIR1 cassette (designated as *tetR’-SSN6*) was integrated into a *his3* strain isogenic to the KSC006 strain [62] by using pST1760 [46]. The *HIS3* marker of *tetR’-SSN6* was replaced with *LEU2* by using pHL3 [63]. Cells were then backcrossed. The mini-AID tag was fused at the N-terminus or both N- and C-termini for the *tel2-aid* or *asa1-aid* construct, respectively. The full-length AID fragment was attached at the C-terminus to construct the *rvb2-aid*. All the AID construct promoters were replaced with the *tetO* cassette. The *MEC1-FLAG* or *TEL1-FLAG* construct (YIp-MEC1-FLAG or YIp-TEL1-FLAG) was converted from *HA-MEC1* or *HA-TEL1* construct [8, 12], respectively, by PCR, NgoMIV restriction digestion and re-ligation. The *MEC1-FLAG* or *TEL1-FLAG* construct was integrated into its own locus after digesting with PshAI or RsrII, respectively. *MEC1-FLAG* cells were as resistant to DNA damaging agents as wild-type cells (S20 Fig). The telomere length of *TEL1-FLAG* cells was very similar to that of wild-type cells (S21 Fig) Gene disruption and C-terminal epitope tagged alleles were generated by PCR-based methods [64–66]. To express HA-tagged Rad53 protein, cells were transformed with YCp-RAD53-HA. The YCp-GAL-FLAG-MEC1 (GAL-FLAG-MEC1) plasmid was generated from YCp-GAL-FLAG-TEL1 (GAL-FLAG-TEL1) after replacing with a PCR-generated NgoMIV-SalI-FLAG-MEC1 fragment. The YCpT-RAD53-HA and the YCp-GAL-FLAG-TEL1 plasmid have been described [55]. The FLAG epitopes were fused to the N-terminus of *MEC1* or *TEL1* at the same location as YIp-MEC1-FLAG or YIp-TEL1-FLAG, respectively. All of the strains used in this study are listed in S1 Table. Oligonucleotides used for plasmid and strain construction are listed in S2 Table.

To deplete AID-tagged protein, cells were treated with 250 μM 3-Indoleacetic acid (IAA; SIGMA) and 10 μg/ml doxycycline (Dox; Enzo Life Science) [45, 46]. Galactose (2%) medium contained glucose (0.5% for Mec1 and 0.3% for Tel1) to express FLAG-Mec1 or -Tel1 from the *GAL1* promoter at the endogenous level, respectively. To monitor Rad53 phosphorylation, cells were incubated with nocodazole (15 μg/ml) for 2 hr to synchronize at G2/M and then treated with 0.1% MMS for 30 min. Cells were cultured at 30°C unless specified. Cells were treated with 10 μg/ml cycloheximide to block translation [67].

### Real-time quantitative reverse transcription PCR

Total RNA was extracted by hot acidic phenol method as described [68]. 20 μg of RNA was treated with 10 units of DNase I (Clontech) in the presence of 20 units of RNaseOUT (Invitrogen) at 37°C for 20 min. DNase I was inactivated by incubation with 25 mM EDTA at 80°C for 2 min. cDNA was synthesized using ProtoScript II First Strand cDNA Synthesis Kit (New England BioLab) according to the manufacturer’s instruction. Real-time PCR was performed as previously described [55]. PCR amplification from *MEC1* and *TEL1* transcript was normalized using that from ACT1 transcript. PCR primers are listed in S2 Table.

### Other methods

Cellular fractionation, immunoprecipitation and immunoblotting were performed as described [55]. Anti-AID antibodies were generated after immunizing rabbits with a synthetic peptide (DGAPYLRKIDLRMYK) or obtained from Dr. Kanemaki.

## Supporting information

S1 Fig DNA flow cytometric analysis of tel2-aid cells. *tel2-aid* cells were grown in the presence (Top) or the absence (Bottom) of IAA and Dox for 6 hr and subjected to flow cytometric analysis [69].
(TIFF)

S2 Fig Effect of IAA and Dox treatment on Mec1 and Tel1 protein expression in aid-untagged cells. *aid-*untagged cells expressing Mec1-FLAG or Tel1-FLAG were cultured with IAA and Dox as in Fig 1C. *asa1-aid* cells were used as a control for IAA/Dox treatment. Cells were subjected to immunoblotting analysis with anti-AID, anti-FLAG or tubulin antibodies.
(TIFF)

S3 Fig Effect of nocodazole treatment Cells expressing Mec1-FLAG or Tel1-FLAG were incubated with (+ Noc) or without nocodazole (- Noc) for 6 hr. Cells were subjected to immunoblotting analysis with anti-FLAG or tubulin antibodies.
(TIFF)

S4 Fig DNA flow cytometric analysis of tel2-aid cells after treatment with nocodazole, IAA and Dox. *tel2-aid* cells expressing Rad53-HA were arrested with nocodazole and then treated with IAA and Dox (+ IAA/Dox; Top) or mock-treated (- IAA/Dox; Middle) as in Fig 1E. Cells were collected before exposure to MMS and subjected to flow cytometric analysis [69]. Untreated cells in asynchronous culture were used as a control (Bottom). Dotted lines indicate the DNA content of 1C and 2C cells. We note that we collected cells before MMS treatment to examine whether cells are arrested after nocodazole treatment. Cells in asynchronous culture accumulate at late S phase after exposure to MMS [70].
(TIFF)

S5 Fig Effect of IAA and Dox treatment on damage-induced Rad53 phosphorylation. Wild-type (KSC1057) and untagged AID host (KSC3413) cells expressing Rad53-HA were analyzed as in Fig 1E.
(TIFF)

S6 Fig Outline of the experimental procedure for Fig 1F. (TIFF)

S7 Fig Outline of the experimental procedure for Fig 1G. (TIFF)

S8 Fig DNA flow cytometric analysis of rvb2-aid cells. *rvb2-aid* cells were grown in the presence (Top) or the absence (Bottom) of IAA and Dox for 6 hr and analyzed as in S1 Fig.
(TIFF)

S9 Fig DNA flow cytometric analysis of rvb2-aid cells after treatment with nocodazole, IAA and Dox. *rvb2-aid* cells expressing Rad53-HA were arrested with nocodazole and then treated with IAA and Dox (+ IAA/Dox; Top) or mock-treated (- IAA/Dox; Middle) as in Fig 2D.
Cells were collected before exposure to MMS and subjected to flow cytometric analysis as in S4 Fig. Untreated cells in asynchronous culture were used as a control (Bottom).
(TIFF)

S10 Fig DNA flow cytometric analysis of asa1-aid cells. *asa1-aid* cells were grown in the presence (Top) or the absence (Bottom) of IAA and Dox for 6 hr and analyzed as in S1 Fig.
(TIFF)

S11 Fig DNA flow cytometric analysis of asa1-aid cells after treatment with nocodazole, IAA and Dox. *asa1-aid* cells expressing Rad53-HA were arrested with nocodazole and then treated with IAA and Dox (+ IAA/Dox; Top) or mock-treated (- IAA/Dox; Middle) as in Fig 4F.
Cells were collected before exposure to MMS and subjected to flow cytometric analysis as in S4 Fig. Untreated cells in asynchronous culture were used as a control (Bottom).
(TIFF)

S12 Fig DNA flow cytometric analysis of *pih1*Δ cells after treatment with nocodazole. Wild-type and *pih1*Δ cells were arrested with nocodazole and incubated at 37 C (Top) or 30 C (Middle) as in Fig 6D, but collected before exposure to MMS.
Cells were then analyzed as in S4 Fig. Untreated cells in asynchronous culture were used as a control (Bottom).
(TIFF)

S13 Fig Effect of cycloheximide treatment on Mec1 and Tel1 protein expression. *MEC1-FLAG* or *TEL1-FLAG* cells were grown at 30°C and treated with cycloheximide. Cells were retained at 30°C or transferred to 37°C or 42°C in the presence of cycloheximide for the indicated times. Cells were then subjected to immunoblotting analysis with anti-FLAG or tubulin antibodies.
(TIFF)

S14 Fig Outline of the experimental procedure for Fig 6E. (TIFF)

S15 Fig Expression of Mec1 and Tel1 in *pih1*Δ cells at 30°C. Wild-type and *pih1*Δ cells, carrying the GAL-FLAG-MEC1 or the GAL-FLAG-TEL1 plasmid, were treated as in Fig 6E but the cultures were retained at 30°C.
Cells are collected and analyzed as in Fig 6E.
(TIFF)

S16 Fig mRNA levels of the FLAG-MEC1 or the FLAG-TEL1 construct. Wild-type and *pih1*Δ cells, carrying the GAL-FLAG-MEC1 or the GAL-FLAG-TEL1 plasmid, were grown in galactose (Galactose) and then transferred to glucose (at 0 hr) as in Fig 6E (see also S14 Fig).
As a negative control, cells were continuously cultured in 2% glucose (Glucose) to repress *GAL-FLAG-MEC1* or *GAL-FLAG-TEL1* expression. Cells were subjected to quantitative PCR analysis to estimate mRNA levels of *FLAG-MEC1* and *FLAG-TEL1*.
(TIFF)

S17 Fig Outline of the experimental procedure for Fig 6F. (TIFF)

S18 Fig Effect of Asa1 depletion on Mec1 and Tel1 protein expression at 30°C. *asa1-aid* cells, carrying the GAL-FLAG-MEC1 or the GAL-FLAG-TEL1 plasmid, were treated as in Fig 6F but the cultures were retained at 30°C.
Cells are collected and analyzed as in Fig 6F.
(TIFF)

S19 Fig mRNA levels of the FLAG-MEC1 or the FLAG-TEL1 construct after transfer from galactose to glucose. *asa1-aid* cells, carrying the GAL-FLAG-MEC1 or the GAL-FLAG-TEL1 plasmid, were grown in galactose (Galactose) and then transferred to glucose with or without IAA/Dox (at 0 hr) as in Fig 6F (see also S17 Fig).
As a negative control, cells were continuously cultured in 2% glucose (Glucose) to repress *GAL-FLAG-MEC1* or *GAL-FLAG-TEL1* expression. Cells were subjected to quantitative PCR analysis to estimate mRNA levels of *FLAG-MEC1* and *FLAG-TEL1*.
(TIFF)

S20 Fig Characterization of the MEC1-FLAG construct. Ten fold serial dilutions of cultures were spotted on yeast extract/peptone/dextrose (YEPD) medium with or without 0.01% MMS or 0.1 mgl/ml hydroxyurea (HU).
Plates were incubated at 30°C for 2 or 3 day. Strains used were the wild type (KSC1516), *mec1Δ* (KSC1561) and *MEC1-FLAG* (YGG487).
(TIFF)

S21 Fig Characterization of the TEL1-FLAG construct. Genomic DNA prepared from cells was digested with *Xho*I and analyzed by Southern blots to monitor the telomere length [55].
The hybridization probe was a DNA fragment containing ~0.9-kb Y′ element and ~120-base pair TG repeat sequence. The bracket shows DNA fragments containing the telomere. Strains used were the wild type (KSC1516), *tel1Δ* (KSC1057) and *TEL1-FLAG* (YHO69).
(TIFF)

S1 Table Strain list. (DOCX)

S2 Table Oligonucleotide list. (DOCX)

## References

[1] Pierce AJ, Stark JM, Araujo FD, Moynahan ME, Berwick M, Jasin M. Double-strand breaks and tumorigenesis. Trends Cell Biol. 2001;11(11):S52–9. . 11684443 10.1016/s0962-8924(01)02149-3

[2] O'Driscoll M, Jeggo PA. The role of double-strand break repair—insights from human genetics. Nat Rev Genet. 2006;7(1):45–54. doi: 10.1038/nrg1746 . 16369571 10.1038/nrg1746

[3] Aguilera A, Garcia-Muse T. Causes of genome instability. Annu Rev Genet. 2013;47:1–32. doi: 10.1146/annurev-genet-111212-133232 . 23909437 10.1146/annurev-genet-111212-133232

[4] Elledge SJ. Cell cycle checkpoints: preventing an identity crisis. Science. 1996;274:1664–72. 8939848 10.1126/science.274.5293.1664

[5] Harper JW, Elledge SJ. The DNA damage response: ten years after. Mol Cell. 2007;28(5):739–45. Epub 2007/12/18. S1097-2765(07)00783-6 [pii] doi: 10.1016/j.molcel.2007.11.015 . 18082599 10.1016/j.molcel.2007.11.015

[6] Paull TT. Mechanisms of ATM Activation. Annu Rev Biochem. 2015 doi: 10.1146/annurev-biochem-060614-034335 . 25580527 10.1146/annurev-biochem-060614-034335

[7] Cimprich KA, Cortez D. ATR: an essential regulator of genome integrity. Nat Rev Mol Cell Biol. 2008;9(8):616–27. doi: 10.1038/nrm2450 ; PubMed Central PMCID: PMC2663384. 18594563 10.1038/nrm2450 PMC2663384

[8] Nakada D, Matsumoto K, Sugimoto K. ATM-related Tel1 associates with double-strand breaks through an Xrs2-dependent mechanism. Genes & Dev. 2003;17:1957–62. 12923051 10.1101/gad.1099003 PMC196250

[9] Falck J, Coates J, Jackson SP. Conserved modes of recruitment of ATM, ATR and DNA-PKcs to sites of DNA damage. Nature. 2005;434(7033):605–11. doi: 10.1038/nature03442 . 15758953 10.1038/nature03442

[10] Paciotti V, Clerici M, Lucchini G, Longhese MP. The checkpoint protein Ddc2, functionally related to *S*. *pombe* Rad26, interacts with Mec1 and is regulated by Mec1-dependent phosphorylation in budding yeast. Genes & Dev. 2000;14:2046–59. 10950868 PMC316858

[11] Rouse J, Jackson SP. *LCD1*: an essential gene involved in checkpoint control and regulation of the *MEC1* signalling pathway in *Saccharomyces cerevisiae*. EMBO J. 2000;19:5793–800. doi: 10.1093/emboj/19.21.5793 11060031 10.1093/emboj/19.21.5801 PMC305794

[12] Wakayama T, Kondo T, Ando S, Matsumoto K, Sugimoto K. Pie1, a protein interacting with Mec1, controls cell growth and checkpoint responses in *Saccharomyces cerevisiae*. Mol Cell Biol. 2001;21:755–64. doi: 10.1128/MCB.21.3.755-764.2001 11154263 10.1128/MCB.21.3.755-764.2001 PMC86667

[13] Cortez D, Guntuku S, Qin J, Elledge SJ. ATR and ATRIP: partners in checkpoint signaling. Science. 2001;294:1713–6. doi: 10.1126/science.1065521 11721054 10.1126/science.1065521

[14] Rouse J, Jackson SP. Lcd1p recruits Mec1p to DNA lesions in vitro and in vivo. Mol Cell. 2002;9:857–69. 11983176 10.1016/s1097-2765(02)00507-5

[15] Zou L, Elledge SJ. Sensing DNA damage through ATRIP recognition of RPA-ssDNA complexes. Science. 2003;300:1542–8. doi: 10.1126/science.1083430 12791985 10.1126/science.1083430

[16] Nakada D, Hirano Y, Sugimoto K. Requirement of the Mre11 complex and exonuclease 1 for activation of the Mec1 signaling pathway. Mol Cell Biol. 2004;24(22):10016–25. doi: 10.1128/MCB.24.22.10016-10025.2004 . 15509802 10.1128/MCB.24.22.10016-10025.2004 PMC525484

[17] Nakada D, Hirano Y, Tanaka Y, Sugimoto K. Role of the C terminus of mec1 checkpoint kinase in its localization to sites of DNA damage. Mol Biol Cell. 2005;16(11):5227–35. doi: 10.1091/mbc.E05-05-0405 . 16148046 10.1091/mbc.E05-05-0405 PMC1266421

[18] Takai H, Wang RC, Takai KK, Yang H, de Lange T. Tel2 regulates the stability of PI3K-related protein kinases. Cell. 2007;131(7):1248–59. doi: 10.1016/j.cell.2007.10.052 . 18160036 10.1016/j.cell.2007.10.052

[19] Hayashi T, Hatanaka M, Nagao K, Nakaseko Y, Kanoh J, Kokubu A, et al Rapamycin sensitivity of the Schizosaccharomyces pombe tor2 mutant and organization of two highly phosphorylated TOR complexes by specific and common subunits. Genes Cells. 2007;12(12):1357–70. doi: 10.1111/j.1365-2443.2007.01141.x . 18076573 10.1111/j.1365-2443.2007.01141.x

[20] Anderson CM, Korkin D, Smith DL, Makovets S, Seidel JJ, Sali A, et al Tel2 mediates activation and localization of ATM/Tel1 kinase to a double-strand break. Genes Dev. 2008;22(7):854–9. doi: 10.1101/gad.1646208 ; PubMed Central PMCID: PMC2279195. 18334620 10.1101/gad.1646208 PMC2279195

[21] Hurov KE, Cotta-Ramusino C, Elledge SJ. A genetic screen identifies the Triple T complex required for DNA damage signaling and ATM and ATR stability. Genes Dev. 2010;24(17):1939–50. doi: 10.1101/gad.1934210 ; PubMed Central PMCID: PMC2932975. 20810650 10.1101/gad.1934210 PMC2932975

[22] Takai H, Xie Y, de Lange T, Pavletich NP. Tel2 structure and function in the Hsp90-dependent maturation of mTOR and ATR complexes. Genes Dev. 2010;24(18):2019–30. doi: 10.1101/gad.1956410 ; PubMed Central PMCID: PMC2939364. 20801936 10.1101/gad.1956410 PMC2939364

[23] Horejsi Z, Takai H, Adelman CA, Collis SJ, Flynn H, Maslen S, et al CK2 phospho-dependent binding of R2TP complex to TEL2 is essential for mTOR and SMG1 stability. Mol Cell. 2010;39(6):839–50. doi: 10.1016/j.molcel.2010.08.037 . 20864032 10.1016/j.molcel.2010.08.037

[24] Ahmed S, Alpi A, Hengartner MO, Gartner A. C. elegans RAD-5/CLK-2 defines a new DNA damage checkpoint protein. Curr Biol. 2001;11(24):1934–44. . 11747819 10.1016/s0960-9822(01)00604-2

[25] Garcia-Muse T, Boulton SJ. Distinct modes of ATR activation after replication stress and DNA double-strand breaks in Caenorhabditis elegans. EMBO J. 2005;24(24):4345–55. doi: 10.1038/sj.emboj.7600896 ; PubMed Central PMCID: PMCPMC1356337. 16319925 10.1038/sj.emboj.7600896 PMC1356337

[26] Shikata M, Ishikawa F, Kanoh J. Tel2 is required for activation of the Mrc1-mediated replication checkpoint. J Biol Chem. 2007;282(8):5346–55. doi: 10.1074/jbc.M607432200 . 17189249 10.1074/jbc.M607432200

[27] Rendtlew Danielsen JM, Larsen DH, Schou KB, Freire R, Falck J, Bartek J, et al HCLK2 is required for activity of the DNA damage response kinase ATR. J Biol Chem. 2009;284(7):4140–7. doi: 10.1074/jbc.M808174200 . 19097996 10.1074/jbc.M808174200

[28] Hoffman KS, Duennwald ML, Karagiannis J, Genereaux J, McCarton AS, Brandl CJ. Saccharomyces cerevisiae Tti2 Regulates PIKK Proteins and Stress Response. G3 (Bethesda). 2016;6(6):1649–59. doi: 10.1534/g3.116.029520 ; PubMed Central PMCID: PMCPMC4889661. 27172216 10.1534/g3.116.029520 PMC4889661

[29] Wellinger RJ, Zakian VA. Everything you ever wanted to know about Saccharomyces cerevisiae telomeres: beginning to end. Genetics. 2012;191(4):1073–105. doi: 10.1534/genetics.111.137851 ; PubMed Central PMCID: PMC3415994. 22879408 10.1534/genetics.111.137851 PMC3415994

[30] Moser BA, Chang YT, Kosti J, Nakamura TM. Tel1ATM and Rad3ATR kinases promote Ccq1-Est1 interaction to maintain telomeres in fission yeast. Nat Struct Mol Biol. 2011;18(12):1408–13. doi: 10.1038/nsmb.2187 ; PubMed Central PMCID: PMCPMC3230746. 22101932 10.1038/nsmb.2187 PMC3230746

[31] Yamazaki H, Tarumoto Y, Ishikawa F. Tel1(ATM) and Rad3(ATR) phosphorylate the telomere protein Ccq1 to recruit telomerase and elongate telomeres in fission yeast. Genes Dev. 2012;26(3):241–6. doi: 10.1101/gad.177873.111 ; PubMed Central PMCID: PMCPMC3278891. 22302936 10.1101/gad.177873.111 PMC3278891

[32] Lee SS, Bohrson C, Pike AM, Wheelan SJ, Greider CW. ATM Kinase Is Required for Telomere Elongation in Mouse and Human Cells. Cell Rep. 2015;13(8):1623–32. doi: 10.1016/j.celrep.2015.10.035 ; PubMed Central PMCID: PMCPMC4663052. 26586427 10.1016/j.celrep.2015.10.035 PMC4663052

[33] Tong AS, Stern JL, Sfeir A, Kartawinata M, de Lange T, Zhu XD, et al ATM and ATR Signaling Regulate the Recruitment of Human Telomerase to Telomeres. Cell Rep. 2015;13(8):1633–46. doi: 10.1016/j.celrep.2015.10.041 ; PubMed Central PMCID: PMCPMC4662887. 26586433 10.1016/j.celrep.2015.10.041 PMC4662887

[34] Runge KW, Zakian VA. TEL2, an essential gene required for telomere length regulation and telomere position effect in Saccharomyces cerevisiae. Mol Cell Biol. 1996;16(6):3094–105. ; PubMed Central PMCID: PMC231304. 8649421 10.1128/mcb.16.6.3094 PMC231304

[35] Benard C, McCright B, Zhang Y, Felkai S, Lakowski B, Hekimi S. The C. elegans maternal-effect gene clk-2 is essential for embryonic development, encodes a protein homologous to yeast Tel2p and affects telomere length. Development. 2001;128(20):4045–55. . 11641227 10.1242/dev.128.20.4045

[36] Lim CS, Mian IS, Dernburg AF, Campisi J. C. elegans clk-2, a gene that limits life span, encodes a telomere length regulator similar to yeast telomere binding protein Tel2p. Curr Biol. 2001;11(21):1706–10. . 11696330 10.1016/s0960-9822(01)00526-7

[37] Stirling PC, Bloom MS, Solanki-Patil T, Smith S, Sipahimalani P, Li Z, et al The complete spectrum of yeast chromosome instability genes identifies candidate CIN cancer genes and functional roles for ASTRA complex components. PLoS Genet. 2011;7(4):e1002057 doi: 10.1371/journal.pgen.1002057 ; PubMed Central PMCID: PMC3084213. 21552543 10.1371/journal.pgen.1002057 PMC3084213

[38] Zhao R, Davey M, Hsu YC, Kaplanek P, Tong A, Parsons AB, et al Navigating the chaperone network: an integrative map of physical and genetic interactions mediated by the hsp90 chaperone. Cell. 2005;120(5):715–27. doi: 10.1016/j.cell.2004.12.024 . 15766533 10.1016/j.cell.2004.12.024

[39] Zhao R, Kakihara Y, Gribun A, Huen J, Yang G, Khanna M, et al Molecular chaperone Hsp90 stabilizes Pih1/Nop17 to maintain R2TP complex activity that regulates snoRNA accumulation. J Cell Biol. 2008;180(3):563–78. doi: 10.1083/jcb.200709061 ; PubMed Central PMCID: PMC2234237. 18268103 10.1083/jcb.200709061 PMC2234237

[40] Pal M, Morgan M, Phelps SE, Roe SM, Parry-Morris S, Downs JA, et al Structural basis for phosphorylation-dependent recruitment of Tel2 to Hsp90 by Pih1. Structure. 2014;22(6):805–18. doi: 10.1016/j.str.2014.04.001 ; PubMed Central PMCID: PMC4058522. 24794838 10.1016/j.str.2014.04.001 PMC4058522

[41] Kakihara Y, Houry WA. The R2TP complex: discovery and functions. Biochimica et biophysica acta. 2012;1823(1):101–7. doi: 10.1016/j.bbamcr.2011.08.016 . 21925213 10.1016/j.bbamcr.2011.08.016

[42] Brown CE, Lechner T, Howe L, Workman JL. The many HATs of transcription coactivators. Trends Biochem Sci. 2000;25(1):15–9. . 10637607 10.1016/s0968-0004(99)01516-9

[43] Shevchenko A, Roguev A, Schaft D, Buchanan L, Habermann B, Sakalar C, et al Chromatin Central: towards the comparative proteome by accurate mapping of the yeast proteomic environment. Genome biology. 2008;9(11):R167 doi: 10.1186/gb-2008-9-11-r167 ; PubMed Central PMCID: PMC2614481. 19040720 10.1186/gb-2008-9-11-r167 PMC2614481

[44] Genereaux J, Kvas S, Dobransky D, Karagiannis J, Gloor GB, Brandl CJ. Genetic evidence links the ASTRA protein chaperone component Tti2 to the SAGA transcription factor Tra1. Genetics. 2012;191(3):765–80. doi: 10.1534/genetics.112.140459 ; PubMed Central PMCID: PMC3389973. 22505622 10.1534/genetics.112.140459 PMC3389973

[45] Nishimura K, Fukagawa T, Takisawa H, Kakimoto T, Kanemaki M. An auxin-based degron system for the rapid depletion of proteins in nonplant cells. Nature methods. 2009;6(12):917–22. doi: 10.1038/nmeth.1401 . 19915560 10.1038/nmeth.1401

[46] Tanaka S, Miyazawa-Onami M, Iida T, Araki H. iAID: an improved auxin-inducible degron system for the construction of a 'tight' conditional mutant in the budding yeast Saccharomyces cerevisiae. Yeast. 2015 doi: 10.1002/yea.3080 . 26081484 10.1002/yea.3080

[47] Kanemaki M, Kurokawa Y, Matsu-ura T, Makino Y, Masani A, Okazaki K, et al TIP49b, a new RuvB-like DNA helicase, is included in a complex together with another RuvB-like DNA helicase, TIP49a. J Biol Chem. 1999;274(32):22437–44. . 10428817 10.1074/jbc.274.32.22437

[48] Lim CR, Kimata Y, Ohdate H, Kokubo T, Kikuchi N, Horigome T, et al The Saccharomyces cerevisiae RuvB-like protein, Tih2p, is required for cell cycle progression and RNA polymerase II-directed transcription. J Biol Chem. 2000;275(29):22409–17. doi: 10.1074/jbc.M001031200 . 10787406 10.1074/jbc.M001031200

[49] Jonsson ZO, Dhar SK, Narlikar GJ, Auty R, Wagle N, Pellman D, et al Rvb1p and Rvb2p are essential components of a chromatin remodeling complex that regulates transcription of over 5% of yeast genes. J Biol Chem. 2001;276(19):16279–88. doi: 10.1074/jbc.M011523200 . 11278922 10.1074/jbc.M011523200

[50] Jha S, Dutta A. RVB1/RVB2: running rings around molecular biology. Mol Cell. 2009;34(5):521–33. doi: 10.1016/j.molcel.2009.05.016 ; PubMed Central PMCID: PMC2733251. 19524533 10.1016/j.molcel.2009.05.016 PMC2733251

[51] Giaever G, Chu AM, Ni L, Connelly C, Riles L, Veronneau S, et al Functional profiling of the Saccharomyces cerevisiae genome. Nature. 2002;418(6896):387–91. doi: 10.1038/nature00935 . 12140549 10.1038/nature00935

[52] Hanna J, Leggett DS, Finley D. Ubiquitin depletion as a key mediator of toxicity by translational inhibitors. Mol Cell Biol. 2003;23(24):9251–61. doi: 10.1128/MCB.23.24.9251-9261.2003 ; PubMed Central PMCID: PMCPMC309641. 14645527 10.1128/MCB.23.24.9251-9261.2003 PMC309641

[53] Wang Y, Liu CL, Storey JD, Tibshirani RJ, Herschlag D, Brown PO. Precision and functional specificity in mRNA decay. Proc Natl Acad Sci U S A. 2002;99(9):5860–5. doi: 10.1073/pnas.092538799 ; PubMed Central PMCID: PMCPMC122867. 11972065 10.1073/pnas.092538799 PMC122867

[54] Munchel SE, Shultzaberger RK, Takizawa N, Weis K. Dynamic profiling of mRNA turnover reveals gene-specific and system-wide regulation of mRNA decay. Mol Biol Cell. 2011;22(15):2787–95. doi: 10.1091/mbc.E11-01-0028 ; PubMed Central PMCID: PMCPMC3145553. 21680716 10.1091/mbc.E11-01-0028 PMC3145553

[55] Ogi H, Goto GH, Ghosh A, Zencir S, Henry E, Sugimoto K. Requirement of the FATC domain of protein kinase Tel1 for localization to DNA ends and target protein recognition. Mol Biol Cell. 2015;26(19):3480–8. doi: 10.1091/mbc.E15-05-0259 ; PubMed Central PMCID: PMC4591692. 26246601 10.1091/mbc.E15-05-0259 PMC4591692

[56] Izumi N, Yamashita A, Iwamatsu A, Kurata R, Nakamura H, Saari B, et al AAA+ proteins RUVBL1 and RUVBL2 coordinate PIKK activity and function in nonsense-mediated mRNA decay. Science signaling. 2010;3(116):ra27 doi: 10.1126/scisignal.2000468 . 20371770 10.1126/scisignal.2000468

[57] Bao Y, Shen X. Chromatin remodeling in DNA double-strand break repair. Curr Opin Genet Dev. 2007;17(2):126–31. doi: 10.1016/j.gde.2007.02.010 . 17320375 10.1016/j.gde.2007.02.010

[58] Hartl FU. Molecular chaperones in cellular protein folding. Nature. 1996;381(6583):571–9. doi: 10.1038/381571a0 . 8637592 10.1038/381571a0

[59] Perry J, Kleckner N. The ATRs, ATMs, and TORs are giant HEAT repeat proteins. Cell. 2003;112(2):151–5. . 12553904 10.1016/s0092-8674(03)00033-3

[60] Inoue H, Sugimoto S, Takeshita Y, Takeuchi M, Hatanaka M, Nagao K, et al CK2 phospho-independent assembly of the Tel2-associated stress-signaling complexes in Schizosaccharomyces pombe. Genes Cells. 2017;22(1):59–70. doi: 10.1111/gtc.12454 . 27935167 10.1111/gtc.12454

[61] Wood V GR, Rajandream MA, Lyne M, Lyne R, Stewart A, Sgouros J, Peat N, Hayles J, Baker S, Basham D, Bowman S, Brooks K, Brown D, Brown S, Chillingworth T, Churcher C, Collins M, Connor R, Cronin A, Davis P, Feltwell T, Fraser A, Gentles S, Goble A, Hamlin N, Harris D, Hidalgo J, Hodgson G, Holroyd S, Hornsby T, Howarth S, Huckle EJ, Hunt S, Jagels K, James K, Jones L, Jones M, Leather S, McDonald S, McLean J, Mooney P, Moule S, Mungall K, Murphy L, Niblett D, Odell C, Oliver K, O'Neil S, Pearson D, Quail MA, Rabbinowitsch E, Rutherford K, Rutter S, Saunders D, Seeger K, Sharp S, Skelton J, Simmonds M, Squares R, Squares S, Stevens K, Taylor K, Taylor RG, Tivey A, Walsh S, Warren T, Whitehead S, Woodward J, Volckaert G, Aert R, Robben J, Grymonprez B, Weltjens I, Vanstreels E, Rieger M, Schäfer M, Müller-Auer S, Gabel C, Fuchs M, Düsterhöft A, Fritzc C, Holzer E, Moestl D, Hilbert H, Borzym K, Langer I, Beck A, Lehrach H, Reinhardt R, Pohl TM, Eger P, Zimmermann W, Wedler H, Wambutt R, Purnelle B, Goffeau A, Cadieu E, Dréano S, Gloux S, Lelaure V, Mottier S, Galibert F, Aves SJ, Xiang Z, Hunt C, Moore K, Hurst SM, Lucas M, Rochet M, Gaillardin C, Tallada VA, Garzon A, Thode G, Daga RR, Cruzado L, Jimenez J, Sánchez M, del Rey F, Benito J, Domínguez A, Revuelta JL, Moreno S, Armstrong J, Forsburg SL, Cerutti L, Lowe T, McCombie WR, Paulsen I, Potashkin J, Shpakovski GV, Ussery D, Barrell BG, Nurse P. The genome sequence of Schizosaccharomyces pombe. nature. 2002;415:871–80. doi: 10.1038/nature724 11859360 10.1038/nature724

[62] Fukunaga K, Hirano Y, Sugimoto K. Subtelomere-binding protein Tbf1 and telomere-binding protein Rap1 collaborate to inhibit localization of the Mre11 complex to DNA ends in budding yeast. Mol Biol Cell. 2012;23(2):347–59. doi: 10.1091/mbc.E11-06-0568 ; PubMed Central PMCID: PMC3258178. 22130795 10.1091/mbc.E11-06-0568 PMC3258178

[63] Cross FR. Marker swap plasmids: convenient tools for budding yeast molecular genetics. Yeast. 1997;13:647–53. doi: 10.1002/(SICI)1097-0061(19970615)13:7<647::AID-YEA115>3.0.CO;2-# 9200814 10.1002/(SICI)1097-0061(19970615)13:7<647::AID-YEA115>3.0.CO;2-#

[64] Goldstein AL, McCusker JH. Three new dominant drug resistance cassettes for gene disruption in Saccharomyces cerevisiae. Yeast. 1999;15(14):1541–53. Epub 1999/10/09. doi: 10.1002/(SICI)1097-0061(199910)15:14<1541::AID-YEA476>3.0.CO;2-K . 10514571 10.1002/(SICI)1097-0061(199910)15:14<1541::AID-YEA476>3.0.CO;2-K

[65] Reid RJ, Lisby M, Rothstein R. Cloning-free genome alterations in Saccharomyces cerevisiae using adaptamer-mediated PCR. Methods Enzymol. 2002;350:258–77. 12073317 10.1016/s0076-6879(02)50968-x

[66] Janke C, Magiera MM, Rathfelder N, Taxis C, Reber S, Maekawa H, et al A versatile toolbox for PCR-based tagging of yeast genes: new fluorescent proteins, more markers and promoter substitution cassettes. Yeast. 2004;21(11):947–62. Epub 2004/08/31. doi: 10.1002/yea.1142 . 15334558 10.1002/yea.1142

[67] Sikorski RS, Boeke JD. In vitro mutagenesis and plasmid shuffling: from cloned gene to mutant yeast. Methods Enzymol. 1991;194:302–18. . 2005795 10.1016/0076-6879(91)94023-6

[68] Sugimoto K, Sakamoto Y, Takahashi O, Matsumoto K. HYS2, an essential gene required for DNA replication in Saccharomyces cerevisiae. Nucleic Acids Res. 1995;23(17):3493–500. Epub 1995/09/11. 5c0085 [pii]. . 7567461 10.1093/nar/23.17.3493 PMC307229

[69] Goto GH, Zencir S, Hirano Y, Ogi H, Ivessa A, Sugimoto K. Binding of Multiple Rap1 Proteins Stimulates Chromosome Breakage Induction during DNA Replication. PLoS Genet. 2015;11(8):e1005283 doi: 10.1371/journal.pgen.1005283 ; PubMed Central PMCID: PMCPMC4532487. 26263073 10.1371/journal.pgen.1005283 PMC4532487

[70] Paulovich AG, Hartwell LH. A checkpoint regulates the rate of progression through S phase in *S*. *cerevisiae* in response to DNA damage. Cell. 1995;82:841–7. 7671311 10.1016/0092-8674(95)90481-6

