# Supplementary figures and images for: Two separate pathways regulate protein stability of ATM/ATR-related protein kinases Mec1 and Tel1 in budding yeast
Source: PLoS Genet. 2017 Aug 21;13(8):e1006873. doi: 10.1371/journal.pgen.1006873 (PMC5578694; doi:10.1371/journal.pgen.1006873)

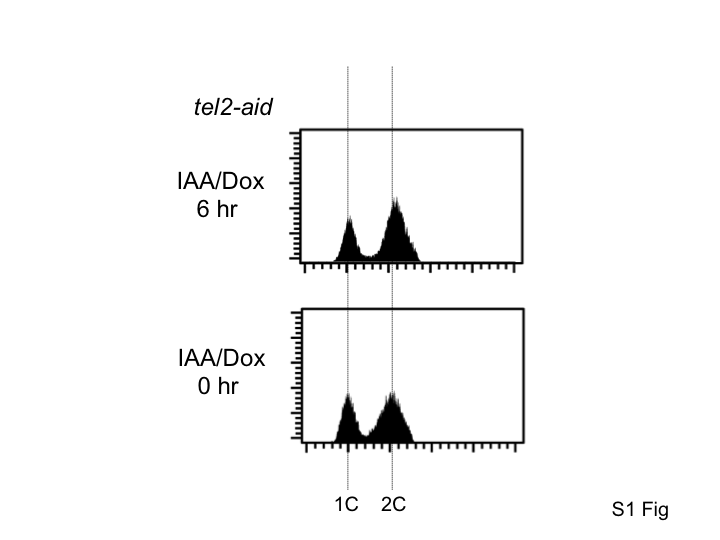

Supplement: S1 Fig — tel2-aid cells were grown in the presence (Top) or the absence (Bottom) of IAA and Dox for 6 hr and subjected to flow cytometric analysis [69]. (TIFF) [file pgen.1006873.s001.tiff]

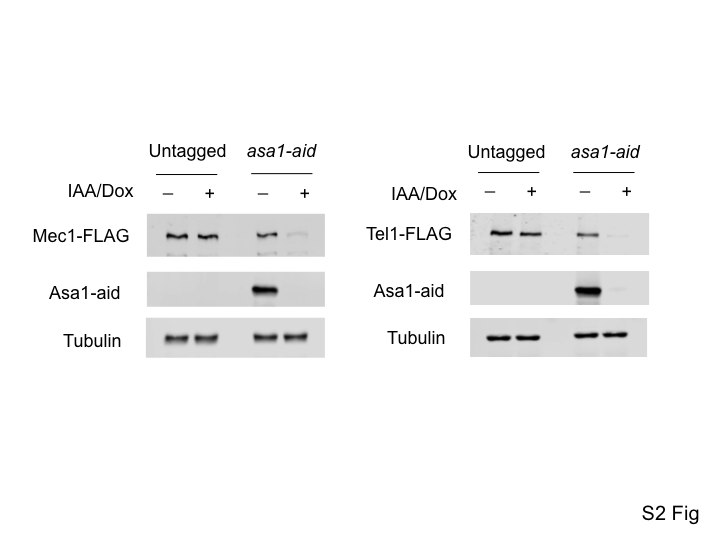

Supplement: S2 Fig — aid-untagged cells expressing Mec1-FLAG or Tel1-FLAG were cultured with IAA and Dox as in Fig 1C. asa1-aid cells were used as a control for IAA/Dox treatment. Cells were subjected to immunoblotting analysis with anti-AID, anti-FLAG or tubulin antibodies. (TIFF) [file pgen.1006873.s002.tiff]

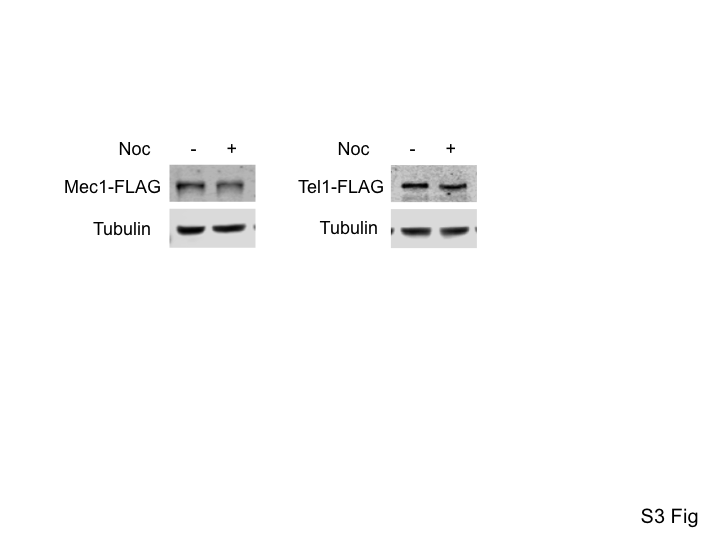

Supplement: S3 Fig — Cells were subjected to immunoblotting analysis with anti-FLAG or tubulin antibodies. (TIFF) [file pgen.1006873.s003.tiff]

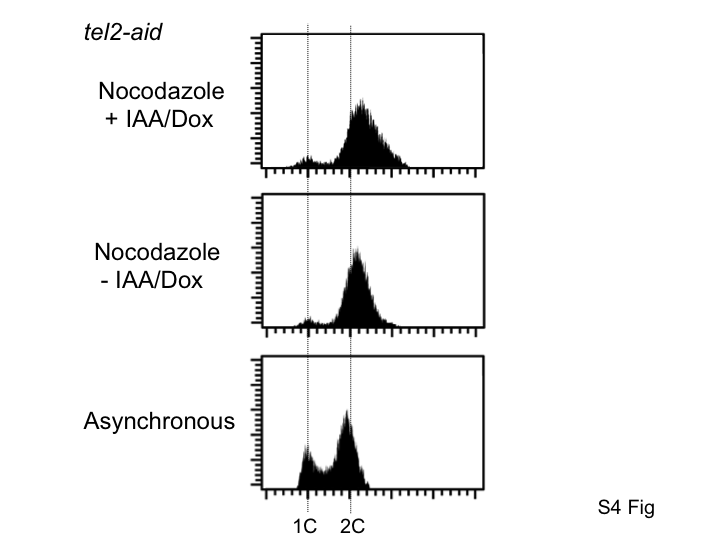

Supplement: S4 Fig — tel2-aid cells expressing Rad53-HA were arrested with nocodazole and then treated with IAA and Dox (+ IAA/Dox; Top) or mock-treated (- IAA/Dox; Middle) as in Fig 1E. Cells were collected before exposure to MMS and subjected to flow cytometric analysis [69]. Untreated cells in asynchronous culture were used as a control (Bottom). Dotted lines indicate the DNA content of 1C and 2C cells. We note that we collected cells before MMS treatment to examine whether cells are arrested after nocodazole treatment. Cells in asynchronous culture accumulate at late S phase after exposure to MMS [70]. (TIFF) [file pgen.1006873.s004.tiff]

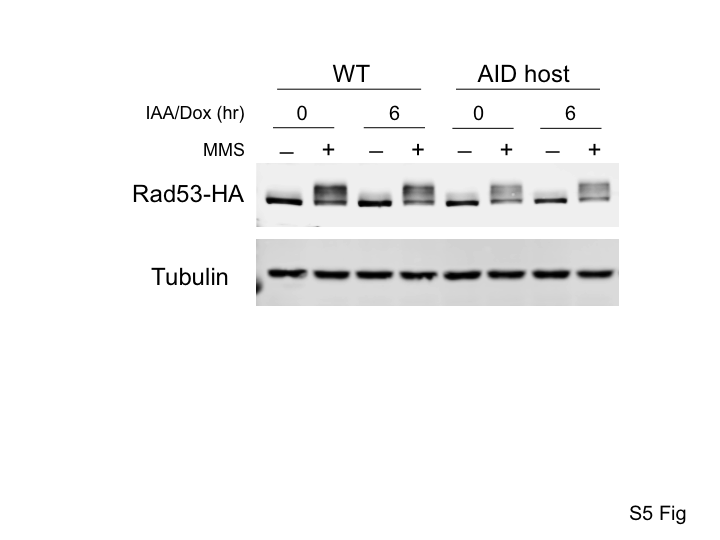

Supplement: S5 Fig — Wild-type (KSC1057) and untagged AID host (KSC3413) cells expressing Rad53-HA were analyzed as in Fig 1E. (TIFF) [file pgen.1006873.s005.tiff]

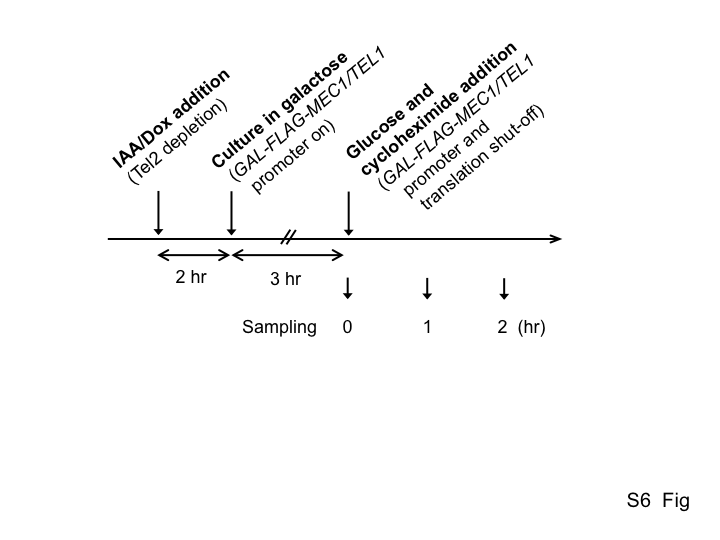

Supplement: S6 Fig — (TIFF) [file pgen.1006873.s006.tiff]

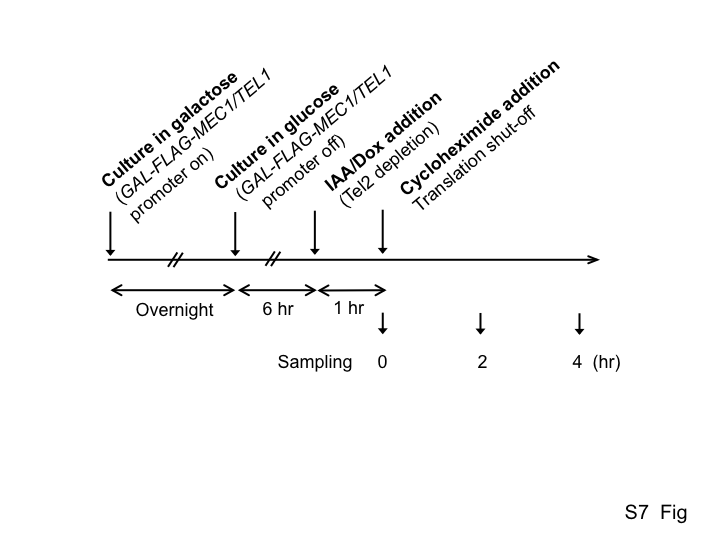

Supplement: S7 Fig — (TIFF) [file pgen.1006873.s007.tiff]

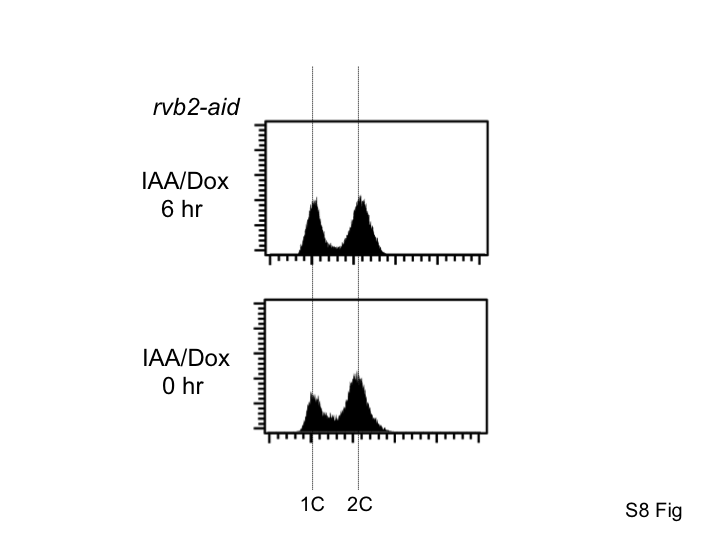

Supplement: S8 Fig — rvb2-aid cells were grown in the presence (Top) or the absence (Bottom) of IAA and Dox for 6 hr and analyzed as in S1 Fig. (TIFF) [file pgen.1006873.s008.tiff]

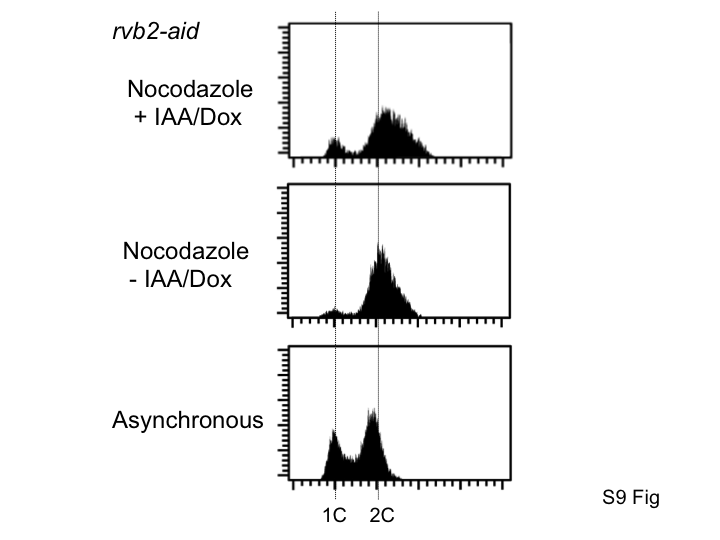

Supplement: S9 Fig — rvb2-aid cells expressing Rad53-HA were arrested with nocodazole and then treated with IAA and Dox (+ IAA/Dox; Top) or mock-treated (- IAA/Dox; Middle) as in Fig 2D. Cells were collected before exposure to MMS and subjected to flow cytometric analysis as in S4 Fig. Untreated cells in asynchronous culture were used as a control (Bottom). (TIFF) [file pgen.1006873.s009.tiff]

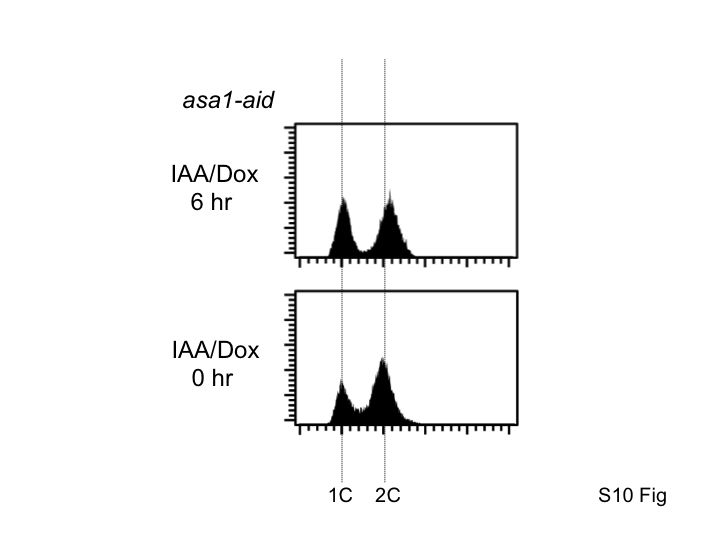

Supplement: S10 Fig — asa1-aid cells were grown in the presence (Top) or the absence (Bottom) of IAA and Dox for 6 hr and analyzed as in S1 Fig. (TIFF) [file pgen.1006873.s010.tiff]

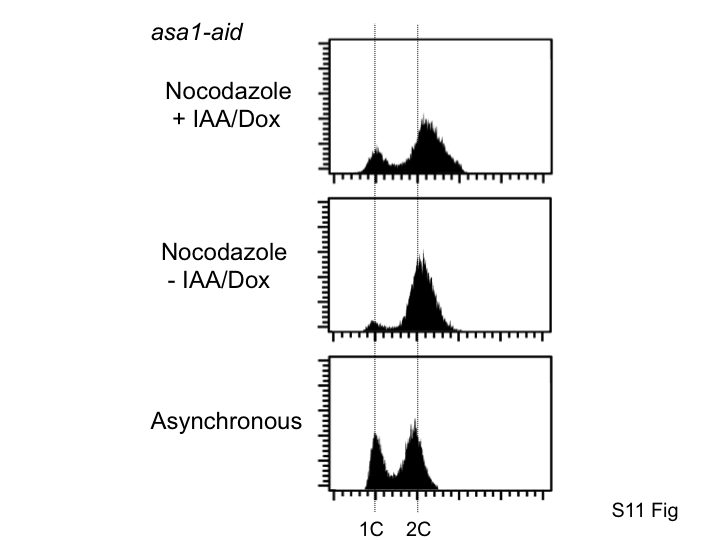

Supplement: S11 Fig — asa1-aid cells expressing Rad53-HA were arrested with nocodazole and then treated with IAA and Dox (+ IAA/Dox; Top) or mock-treated (- IAA/Dox; Middle) as in Fig 4F. Cells were collected before exposure to MMS and subjected to flow cytometric analysis as in S4 Fig. Untreated cells in asynchronous culture were used as a control (Bottom). (TIFF) [file pgen.1006873.s011.tiff]

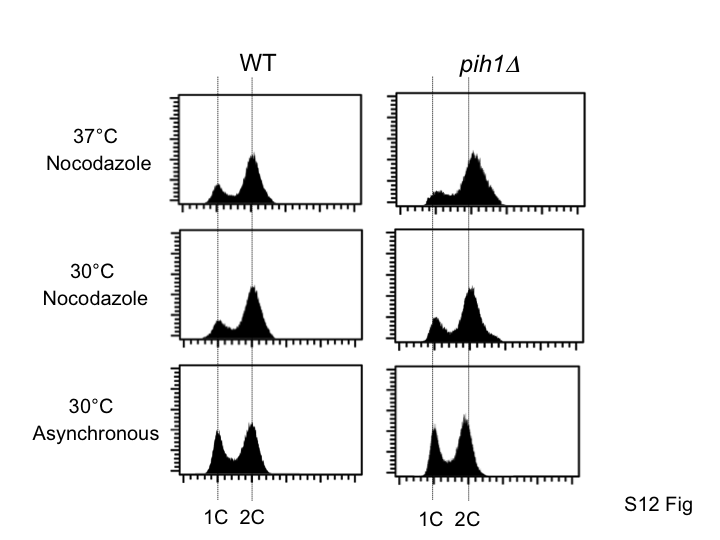

Supplement: S12 Fig — Wild-type and pih1Δ cells were arrested with nocodazole and incubated at 37 C (Top) or 30 C (Middle) as in Fig 6D, but collected before exposure to MMS. Cells were then analyzed as in S4 Fig. Untreated cells in asynchronous culture were used as a control (Bottom). (TIFF) [file pgen.1006873.s012.tiff]

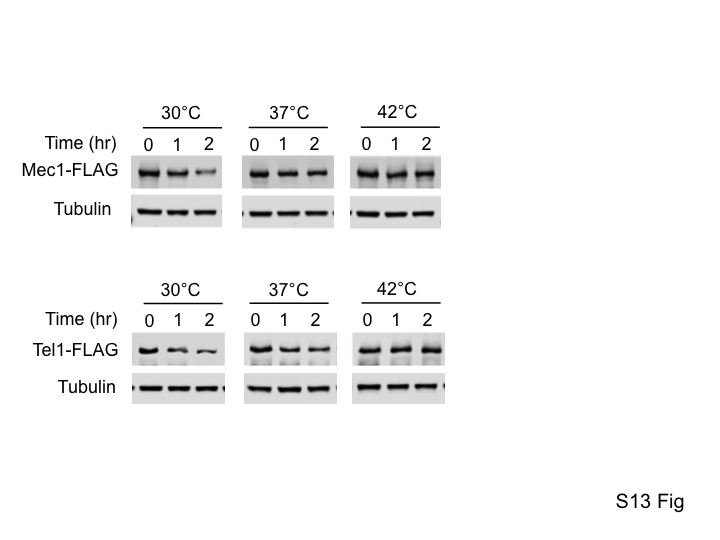

Supplement: S13 Fig — MEC1-FLAG or TEL1-FLAG cells were grown at 30°C and treated with cycloheximide. Cells were retained at 30°C or transferred to 37°C or 42°C in the presence of cycloheximide for the indicated times. Cells were then subjected to immunoblotting analysis with anti-FLAG or tubulin antibodies. (TIFF) [file pgen.1006873.s013.tiff]

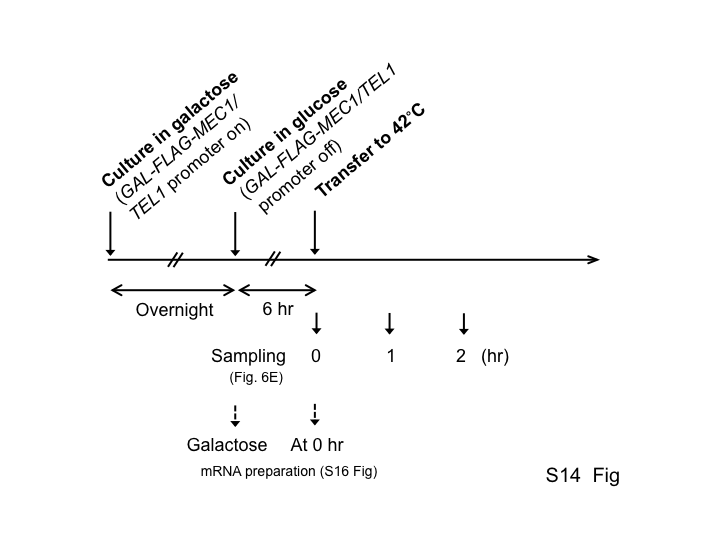

Supplement: S14 Fig — (TIFF) [file pgen.1006873.s014.tiff]

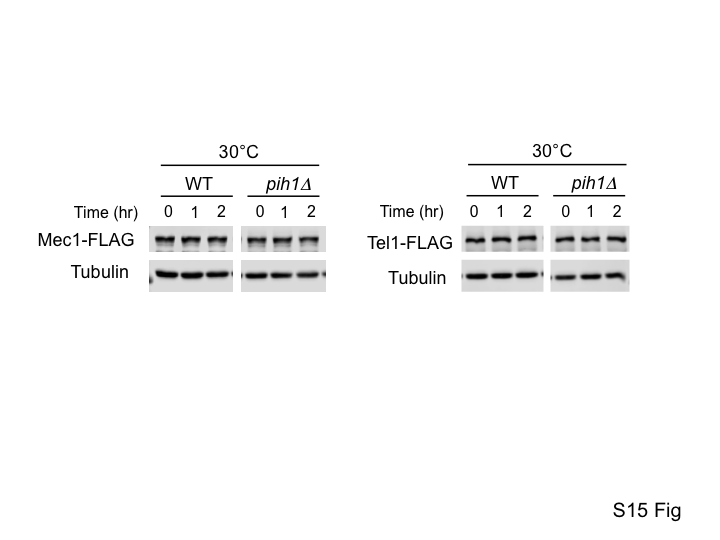

Supplement: S15 Fig — Wild-type and pih1Δ cells, carrying the GAL-FLAG-MEC1 or the GAL-FLAG-TEL1 plasmid, were treated as in Fig 6E but the cultures were retained at 30°C. Cells are collected and analyzed as in Fig 6E. (TIFF) [file pgen.1006873.s015.tiff]

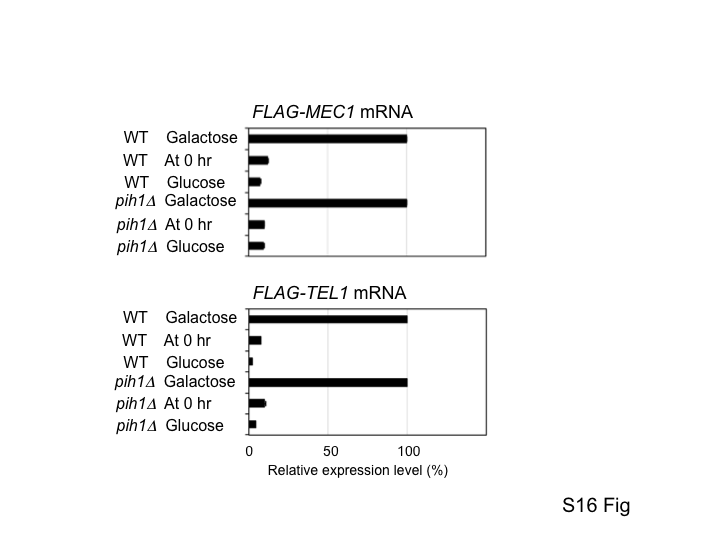

Supplement: S16 Fig — Wild-type and pih1Δ cells, carrying the GAL-FLAG-MEC1 or the GAL-FLAG-TEL1 plasmid, were grown in galactose (Galactose) and then transferred to glucose (at 0 hr) as in Fig 6E (see also S14 Fig). As a negative control, cells were continuously cultured in 2% glucose (Glucose) to repress GAL-FLAG-MEC1 or GAL-FLAG-TEL1 expression. Cells were subjected to quantitative PCR analysis to estimate mRNA levels of FLAG-MEC1 and FLAG-TEL1. (TIFF) [file pgen.1006873.s016.tiff]

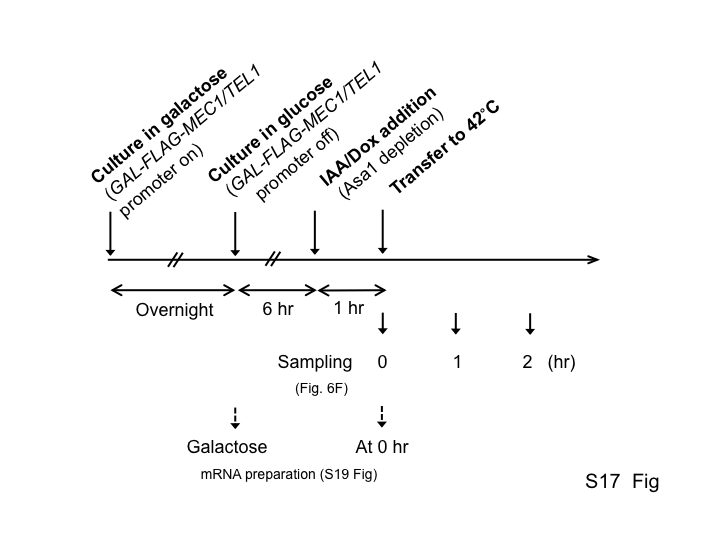

Supplement: S17 Fig — (TIFF) [file pgen.1006873.s017.tiff]

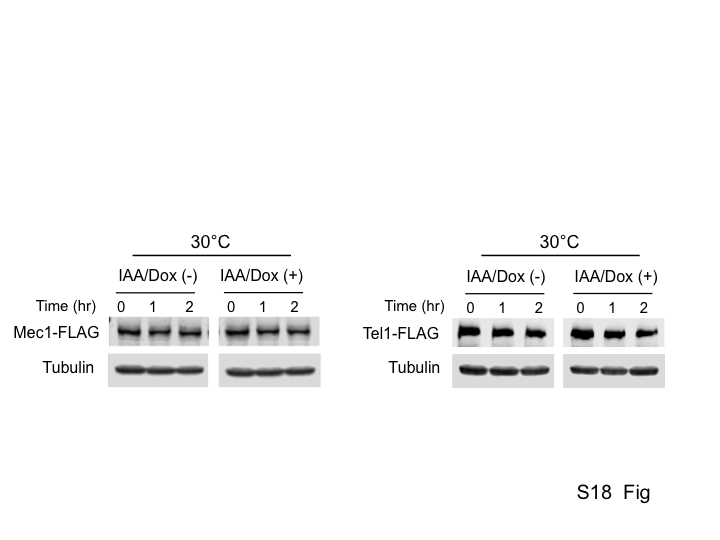

Supplement: S18 Fig — asa1-aid cells, carrying the GAL-FLAG-MEC1 or the GAL-FLAG-TEL1 plasmid, were treated as in Fig 6F but the cultures were retained at 30°C. Cells are collected and analyzed as in Fig 6F. (TIFF) [file pgen.1006873.s018.tiff]

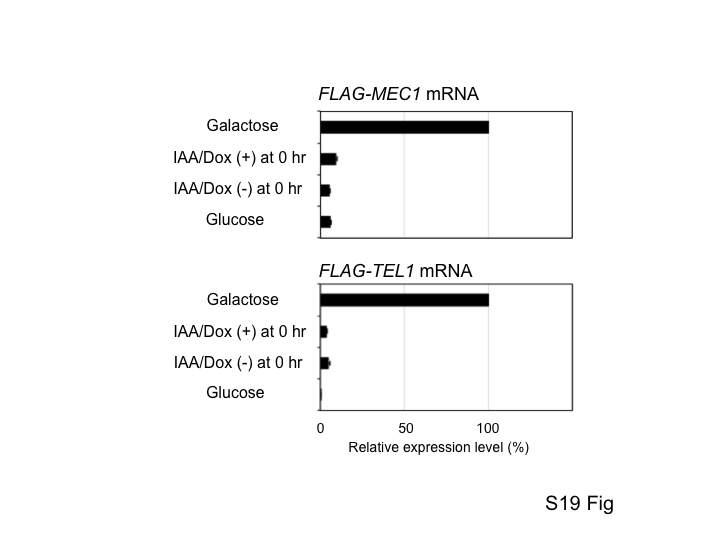

Supplement: S19 Fig — asa1-aid cells, carrying the GAL-FLAG-MEC1 or the GAL-FLAG-TEL1 plasmid, were grown in galactose (Galactose) and then transferred to glucose with or without IAA/Dox (at 0 hr) as in Fig 6F (see also S17 Fig). As a negative control, cells were continuously cultured in 2% glucose (Glucose) to repress GAL-FLAG-MEC1 or GAL-FLAG-TEL1 expression. Cells were subjected to quantitative PCR analysis to estimate mRNA levels of FLAG-MEC1 and FLAG-TEL1. (TIFF) [file pgen.1006873.s019.tiff]

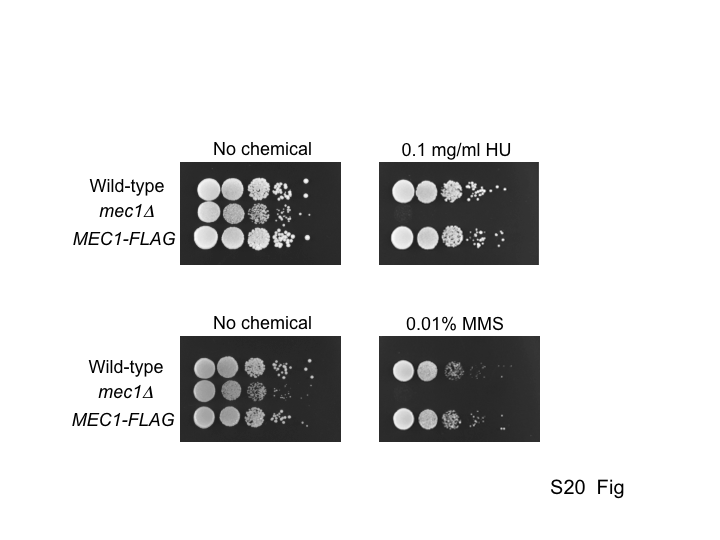

Supplement: S20 Fig — Ten fold serial dilutions of cultures were spotted on yeast extract/peptone/dextrose (YEPD) medium with or without 0.01% MMS or 0.1 mgl/ml hydroxyurea (HU). Plates were incubated at 30°C for 2 or 3 day. Strains used were the wild type (KSC1516), mec1Δ (KSC1561) and MEC1-FLAG (YGG487). (TIFF) [file pgen.1006873.s020.tiff]

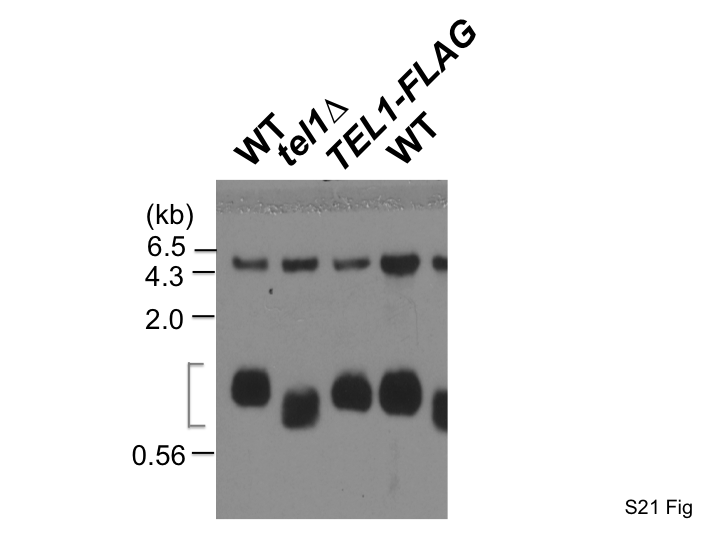

Supplement: S21 Fig — Genomic DNA prepared from cells was digested with XhoI and analyzed by Southern blots to monitor the telomere length [55]. The hybridization probe was a DNA fragment containing ~0.9-kb Y′ element and ~120-base pair TG repeat sequence. The bracket shows DNA fragments containing the telomere. Strains used were the wild type (KSC1516), tel1Δ (KSC1057) and TEL1-FLAG (YHO69). (TIFF) [file pgen.1006873.s021.tiff]
