# Supplementary material for: Two separate pathways regulate protein stability of ATM/ATR-related protein kinases Mec1 and Tel1 in budding yeast
Source: PLoS Genet. 2017 Aug 21;13(8):e1006873. doi: 10.1371/journal.pgen.1006873 (PMC5578694; doi:10.1371/journal.pgen.1006873)
Supplement: S1 Table — (DOCX) [file pgen.1006873.s022.docx]

**Table S1. Strains used in this study**

___________________________________________________________________________________

Strain Genotype

____________________________________________________________________________________________

KSC1057 *MAT***a** *tel1∆::LEU2*

KSC1516 *MAT***a***-inc*

KSC1560 *MAT***a***-inc sml1∆::LEU2*

KSC1561 *MAT***a***-inc mec1∆::LEU2 sml1∆::LEU2*

KSC3394 *MAT***a** *tetR’-SSN6::HIS3 tetO-tel2-aid*::*HphMX*

KSC3413 *MAT***a** *tetR’-SSN6::LEU2*

YHO167 *MAT***a** *tetR’-SSN6::LEU2 MEC1-FLAG::TRP1*

YHO164 *MAT***a** *tetR’-SSN6::LEU2 TEL1-FLAG::TRP1*

YGG599 *MAT***a** *tetR’-SSN6::HIS3 tetO-tel2-aid::HphMX MEC1-FLAG::TRP1*

YGG598 *MAT***a** *tetR’-SSN6::HIS3 tetO-tel2-aid::HphMX TEL1-FLAG::TRP1*

YHO246 *MAT***a** *tetR’-SSN6::LEU2 tetO-rvb2-aid::HphMX-KanMX*

YHO348 *MAT***a** *tetR’-SSN6::LEU2 tetO-rvb2-aid::HphMX-KanMX MEC1-FLAG::TRP1*

YHO331 *MAT***a** *tetR’-SSN6::LEU2 tetO-rvb2-aid::HphMX-KanMX TEL1-FLAG::TRP1*

YHO97  *MAT***a***-inc pih1∆::HphMX*

YGG487  *MAT***a***-inc MEC1-FLAG::TRP1*

YHO98  *MAT***a***-inc MEC1-FLAG::TRP1 pih1∆::HphMX*

YHO69  *MAT***a***-inc TEL1-FLAG::TRP1*

YHO99  *MAT***a***-inc TEL1-FLAG::TRP1 pih1∆::HphMX*

YHO104 *MAT***a***-inc RVB1*-*myc*::*KanMX*

YHO131 *MAT***a***-inc TEL2*-*HA*::*TRP1 RVB1*-*myc*::*KanMX*

YHO178 *MAT***a***-inc TEL2*-*HA*::*TRP1 RVB1*-*myc*::*KanMX* *pih1∆*::*HphMX*

YHO478  *MAT***a***-inc PIH1-9myc::KanMX*

YHO481  *MAT***a***-inc PIH1-9myc::KanMX TEL2-HA::TRP1*

YHO250  *MAT***a***-inc ASA1-9myc::KanMX*

YHO287  *MAT***a***-inc ASA1-9myc::KanMX MEC1-HA::TRP1*

YHO291  *MAT***a***-inc ASA1-9myc::KanMX TEL1-HA::TRP1*

YHO108  *MAT***a** *tetR’-SSN6::LEU2 tetO-asa1-aid::HphMX-NatMX*

YHO169  *MAT***a** *tetR’-SSN6::LEU2 tetO-asa1-aid::HphMX-NatMX MEC1-FLAG::TRP1*

YHO166  *MAT***a** *tetR’-SSN6::LEU2 tetO-asa1-aid::HphMX-NatMX TEL1-FLAG::TRP1*

YHO160  *MAT***a** *tetR’-SSN6::LEU2 tetO-asa1-aid::HphMX-NatMX TEL2-HA::TRP1 RVB1-myc::KanMX*

YHO323 *MAT***a** *tetR’-SSN6::LEU2 tetO-rvb2-aid::HphMX-KanMX TEL2-HA::TRP1 ASA1-myc::KanMX*

YHO209 *MAT***a** *tetR’-SSN6*::*HIS3 tetO-tel2-aid*::*HphMX ASA1-6HA::TRP1 RVB1-myc::KanMX*

YHO518 *MAT***a***-inc ASA1-6HA::TRP1 PIH1-myc::KanMX*

YHO304 *MAT****a*** *tetR’-SSN6::LEU2 tetO-asa1-aid::HphMX-NatMX MEC1-HA::URA3 TEL2-FLAG::KanMX*

YHO302 *MAT****a*** *tetR’-SSN6::LEU2 tetO-asa1-aid::HphMX-NatMX TEL1-HA::URA3 TEL2-FLAG::KanMX*

YHO95  *MAT***a***-inc TEL2-6HA::TRP1*

YHO132  *MAT***a***-inc ASA1-6HA::TRP1*

_______________________________________________________________________________

All the strains are isogenic to KSC006 (*MAT***a,** *ade1*, *his2*, *leu2*, *trp1*, *ura3*).
