# Supplementary material for: Two separate pathways regulate protein stability of ATM/ATR-related protein kinases Mec1 and Tel1 in budding yeast
Source: PLoS Genet. 2017 Aug 21;13(8):e1006873. doi: 10.1371/journal.pgen.1006873 (PMC5578694; doi:10.1371/journal.pgen.1006873)
Supplement: S2 Table — (DOCX) [file pgen.1006873.s023.docx]

**Table S2. List of oligonucleotides used in this study**

| Name | Sequence (5’ – 3’) |  |
| --- | --- | --- |
| KS3042 | GGAGaATTCGGACTAATCTCATTCAGC | *tel2-aid* |
| KS3044 | cgtatgtgaatgctggtcgctatactgCGGCAACCTGTCCAC | *tel2-aid* |
| KS3049 | TCATGTATTTGACTGCTGTCAAGCCCTTGTTTCAGCGTTTCTAAAACCATggaacctccTCTAGGTACAAG | *tel2-aid* |
| KS3273 | CGGTGTCATAAGACAGGAC | *rvb2-aid* |
| KS3274 | cgtatgtgaatgctggtcgctatactgCTTGTGCTAGACTGCTAGAG | *rvb2-aid* |
| KS3275 | AACGACTTTAAATCTGATGTTTCATTTGGATCACTAGTTTGAATCGACATagcactcttttctttagcaccc | *rvb2-aid* |
| KS3248 | GGAAGTTGAACTGTCATCCG | *rvb2-aid* and *RVB2-HA* |
| KS3249 | gtcgacctgcagcgtacgTTCCGTAGTATCCATGGCATC | *rvb2-aid* and *RVB2-HA* |
| KS3250 | gGTTCTGAACGTGCTGGCCAACTGCTGCCTATTTTAGGTGTatacgactcactataggg | *rvb2-aid* and *RVB2-HA* |
| KS3225 | GATAGTACTAGACCACATAGAGC | *asa1-aid* |
| KS3226 | cgtatgtgaatgctggtcgctatactgTAATCGACGCTCGCCTTGAC | *asa1-aid* |
| KS3227 | GATAAAGTCAACGTTCTCTTCAGGATTATCTCATTACTAAAGCCTCTCATggaacctccTCTAGGTACAAG | *asa1-aid* |
| KS3230 | TGCGGACTTCAGAAGTGGAG | *asa1-aid* |
| KS3231 | gtcgacctgcagcgtacgTATTTTATTGAGCTTTATGGTACCATCTTC | *asa1-aid* |
| KS3232 | AACTCCCTTTAACATTCTTATGACCATGATACATCGCACtaatacgactcactataggg | *asa1-aid* |
| KS2872 | gaagccggcgattataaagatgacgatgataaggaatcacacgtcaaatatc | *MEC1-FLAG* |
| KS3120 | ttcgccggccttatcatcgtcatctttataatcTTCCATGCAGTCTTGTGGGC | *MEC1-FLAG* |
| KS1583 | gaagccggcgattataaagatgacgatgataagGAGGATCATGGGATTGTAGAAA | *TEL1-FLAG* |
| KS1692 | ttcgccggccttatcatcgtcatctttataatcCTCCATCGTCCTTGACGTTAAAGTATAGAGGT | *TEL1-FLAG* |
| KS3234 | GTTGGAAGGTACCAGTGGC | *pih1∆* |
| KS3235 | gtgtcacctaaatcgtatgtgGTCTCAATAAGAAATCGGCC | *pih1∆* |
| KS3236 | GAAGATTCACGATGTTTTGGGCTACAGTGTGAGTGAATACGTAGCACAtaatacgactcactataggg | *pih1∆* |
| KS2789 | ATCATATAATTAGTTCAGCCATAGAGGAAGGAATTTCTCTCAATAAAGGTcgtacgctgcaggtcgac | *TEL2-HA* |
| KS2790 | AAATAATATGCTGTGCATTAAATACTAACGACGCTGACAGAGAACGCACTAATACGACTCACTATAGGG | *TEL2-HA* |
| KS3244 | TGATCAAGGTGTCGCAGAAC | *RVB1-myc* |
| KS3245 | gtcgacctgcagcgtacgCAAATAATTTGCGGAAGTTTCt | *RVB1-myc* |
| KS3246 | CAGCAACAACGATGCATAATTGGCATCTAGCCCCAATGACGatacgactcactataggg | *RVB1-myc* |
| KS3240 | ACAGTGCGTCCTATGCTCTG | *TTI1-myc* |
| KS3241 | gtcgacctgcagcgtacgATTTATATTTCTTATTTTCCACACGatatcc | *TTI1-myc* |
| KS3242 | CTTATTGTAATCACTATAGTCACTGGTGTGAACAATGTGAGCatacgactcactataggg | *TTI1-myc* |
| KS3255 | TGTACATCTCCAGAGGCTG | *TTI2-myc* |
| KS3256 | gtcgacctgcagcgtacgTGAGAACTCAAAAAGTAACTGAAACCG | *TTI2-myc* |
| KS3116 | GAATATTTTAATATATGTCCAAGGTTTCTTTAAAAGGGATTCTTCAGCAtaatacgactcactataggg | *TTI2-myc* |
| KS2023 | agtaccacggaccttatacc | *MEC1 qRT-PCR* |
| KS3280 | GCGCACTTCTCGCATAGG | *MEC1 qRT-PCR* |
| KS947 | GAGATTCTGAAGTACTAAAGGCAC | *TEL1 qRT-PCR* |
| KS3279 | GGATATGTTGGTGTCCTTATTGG | *TEL1 qRT-PCR* |
| KS3281 | CAAGGTATCATGGTCGGTATG | *ACT1 qRT-PCR* |
| KS3282  KS3544  KS3545  KS521 | AGTAACACCATCACCGGAATC  GATGACGATGATAAGGCCG  GTTCCCGTTGGAATCCTTC  CTGTTCTCACTAAGTGATAGCTTGT | *ACT1 qRT-PCR*  *FLAG qRT-PCR*  *MEC1-FLAG*  *qRT-PCR*  *TEL1-FLAG qRT-PCR* |
